# COL11A1 promotes lung adenocarcinoma progression via PI3K/AKT/mTOR pathway: mechanistic insights and development of a COL11A1-related prognostic signature

**DOI:** 10.3389/fonc.2026.1748723

**Published:** 2026-02-27

**Authors:** Weijie Wu, Yuhang Chen, Chenlu Gong, Yujie Xiao, Caixia Zhang, Chonghua Hao, Xiaoyan Li, Guoyin Li, Xi Zhang

**Affiliations:** 1 Department of Oncology, The Third Xiangya Hospital of Central South University, Central South University, Changsha, China; 2 College of Life Science and Agronomy, Zhoukou Normal University, Zhoukou, China; 3 Department of Clinical Laboratory, Heping Branch, Shanxi Provincial People’s Hospital, Taiyuan, China

**Keywords:** COL11A1, lung adenocarcinoma (LUAD), PI3K/AKT/mTOR signaling pathway, prognostic model, twist

## Abstract

**Background:**

Lung cancer is the leading cause of cancer-related deaths worldwide. Lung adenocarcinoma (LUAD) accounts for 40% of all lung cancer cases, with a 5-year survival rate of less than 20%. Delayed diagnosis, high recurrence rate, and drug resistance are the main factors contributing to its poor prognosis. Collagen type XI alpha 1 chain (COL11A1) has been shown to promote tumor invasion and metastasis in various malignant tumors; however, its expression regulatory mechanism and biological function in LUAD remain unclear. This study aimed to investigate the effect of COL11A1 on LUAD progression and its underlying molecular mechanism, and to construct a relevant prognostic evaluation model, thereby providing a basis for the diagnosis and treatment of LUAD.

**Methods:**

The expression characteristics of COL11A1 were determined through multi-omics analysis of public datasets (TCGA_LUAD, GSE series) and clinical specimens. Western blot, chromatin immunoprecipitation (ChIP), and luciferase reporter gene assays were used to elucidate the regulatory mechanism of COL11A1. COL11A1-related risk score (CRRS) and a nomogram were constructed based on LASSO-Cox regression analysis, followed by validation in multiple cohorts.

**Results:**

COL11A1 expression was significantly upregulated in LUAD tissues, and its high expression was closely associated with poor prognosis of LUAD patients, with an area under the receiver operating characteristic curve (AUC) > 0.93. *In vitro* and *in vivo* experiments confirmed that COL11A1 could promote the proliferation of LUAD cells by activating the PI3K/AKT/mTOR signaling pathway. The transcription factor TWIST positively regulated COL11A1 expression by directly binding to its promoter region. The CRRS constructed based on 7 core genes successfully stratified patients in both the training and validation cohorts into high-risk and low-risk groups, with significant differences in survival rates between the two groups. Additionally, CRRS was correlated with drug sensitivity. The nomogram integrating CRRS and clinical variables effectively predicted the 1-year, 3-year, and 5-year survival rates of LUAD patients (AUC > 0.71).

**Conclusions:**

COL11A1 acts as an oncogene in LUAD. Its expression is transcriptionally activated by the transcription factor TWIST, and it exerts pro-tumor effects by activating the PI3K/AKT/mTOR signaling pathway. CRRS and the nomogram provide potential references for prognostic evaluation and precision treatment of LUAD patients.

## 1 Introduction

Lung cancer ranks as the second most common malignant tumor globally and has long remained the leading cause of cancer-related deaths (1). As the predominant subtype of lung cancer, lung adenocarcinoma (LUAD) accounts for approximately 40% of all lung cancer cases (2). Regrettably, the 5-year survival rate of LUAD patients is less than 20%, a grim reality primarily attributed to difficulties in early diagnosis, high postoperative recurrence rates, and frequent occurrence of chemotherapy resistance (3). Despite the continuous advancement of diagnostic technologies and therapeutic strategies for LUAD in recent years, the overall survival outcomes of patients have not been significantly improved. Therefore, in-depth exploration of the core molecular mechanisms underlying the initiation and progression of LUAD, identification of prognostic biomarkers and therapeutic targets with clinical value, and establishment of individualized diagnosis and treatment systems have become the current research focus and urgent needs in this field.

The COL11A1 gene encodes the α1 chain of type XI collagen, which, as a critical component of the extracellular matrix (ECM), is involved in regulating multiple physiological processes such as cell proliferation, differentiation, and migration (4–6). Type XI collagen is inherently a heterotrimeric structure that is primarily expressed in the chondrocyte ECM under physiological conditions, playing a vital role in skeletal morphogenesis (7). In recent years, a growing body of studies has confirmed that dysregulated COL11A1 function is closely associated with the progression of various malignant tumors: in cancers including glioma (8), neuroblastoma (9), and oral squamous cell carcinoma (10), aberrantly high COL11A1 expression significantly enhances the invasive and metastatic capabilities of tumor cells. Additionally, COL11A1 can further promote tumor progression by regulating the infiltration and activation of immune cells in the tumor microenvironment (10–12). In pancreatic cancer, COL11A1 drives a positive feedback loop to induce the transformation of normal fibroblasts into cancer-associated fibroblasts (CAFs), thereby facilitating tumor cell invasion (13). In breast cancer, high COL11A1 expression is closely linked to malignant tumor progression, and its expression is regulated by the CDX2/let-7b axis (14). In lung adenocarcinoma (LUAD), COL11A1 has been reported to promote tumor initiation and development via the PI3K/Akt/GSK-3β regulatory axis (15).

Although existing studies have suggested an association between COL11A1 and lung cancer (24), critical questions remain unresolved regarding its role in LUAD, the specific subtype of lung cancer. Specifically, its expression regulatory mechanisms (e.g., upstream transcription factors), downstream pro-tumor signaling pathways, and potential as a clinically applicable prognostic biomarker have not been systematically elucidated. Obvious gaps persist in the current research: Does COL11A1 exhibit specific expression characteristics in LUAD? How does it regulate the malignant phenotype of LUAD cells? Can a reliable clinical prognostic evaluation tool be constructed based on COL11A1?

Against this research background, the present study first clarified the expression characteristics and clinical significance of COL11A1 in LUAD through multi-omics analysis. Subsequently, its biological functions were validated using *in vitro* cell experiments and *in vivo* xenograft models. Furthermore, we comprehensively explored its upstream regulatory factors and downstream signaling pathways, and constructed a COL11A1-related risk score (CRRS) along with a nomogram. This study aims to identify a novel molecular target for LUAD and develop a practical prognostic evaluation tool, thereby advancing the precision diagnosis and treatment of this disease.

## 2 Materials and methods

### 2.1 Acquisition and processing of public data

The TCGA-LUAD dataset was downloaded from The Cancer Genome Atlas (TCGA) database (https://portal.gdc.cancer.gov), and the GSE43458, GSE68465, GSE10072, GSE31210, GSE72094, and GSE37745 datasets were retrieved from the Gene Expression Omnibus (GEO) database (https://ncbi.nlm.nih.gov/gds).

Inclusion criteria:

Cases pathologically confirmed as lung adenocarcinoma (LUAD).

Datasets containing complete gene expression profiles and clinical prognostic information (e.g., overall survival, tumor stage).

Data preprocessing was performed following the standardized protocol previously published by our research team (16, 17). All the aforementioned datasets are publicly available resources, thus no additional ethical approval was required for their use.

### 2.2 Unsupervised clustering analysis

Clustering analysis was conducted using R software (Version 4.1.0):

Consensus Clustering (CC): The “ConsensusClusterPlus” package was employed for consensus clustering. The number of clusters (K) was set to range from 2 to 10. The optimal number of clusters was determined by analyzing the consensus matrix heatmap, cumulative distribution function (CDF) curve, and ΔCDF values.

Nonnegative Matrix Factorization (NMF) Clustering: The “NMF” package was used for nonnegative matrix factorization clustering. The cophenetic coefficient was utilized to evaluate the stability of clustering, and based on this, the optimal number of subgroups was identified.

The input data for clustering analysis was the standardized expression matrix of COL11A1 and its co-expressed genes.

### 2.3 Construction of COL11A1-related risk score

The TCGA-LUAD cohort was used as the training set, and the GSE68465, GSE72094, and GSE31210 datasets served as external validation sets. The construction process was as follows:

Screening of co-expressed genes: The “corrplot” package in R software was used to calculate the Pearson correlation coefficients between COL11A1 and other genes in the TCGA-LUAD dataset. Genes with a correlation coefficient ≥ 0.5 and P ≤ 0.001 were selected, resulting in a total of 182 COL11A1 co-expressed genes (**Supplementary Table 1**).

Screening of differentially expressed genes (DEGs): Combining the results of consensus clustering (CC) and nonnegative matrix factorization (NMF) clustering, patients in the TCGA-LUAD cohort were divided into two subgroups. The “limma” package was applied to screen DEGs between the two subgroups with the criteria of |logFC| ≥ 2 and adj.P value ≤ 0.01. Finally, 81 core DEGs related to prognosis were obtained (**Supplementary Table 2**).

Construction of risk score model: The “glmnet” package was used to perform LASSO-Cox regression analysis on the 81 DEGs. The optimal penalty parameter λ was determined through 10-fold cross-validation, and 7 core prognostic genes and their corresponding coefficients were screened out. The formula for calculating the risk score was: score = ∑i [Coefficient (Gene i) × Expression (Gene i)] (18).

### 2.4 Drug sensitivity analysis

In R 4.1.0 software, gene expression data were normalized using the “limma” package, and the half-maximal inhibitory concentration (IC50) of chemotherapeutic drugs in patients was predicted using the “pRRophetic” package. The drug sensitivity data were derived from the Genomics of Drug Sensitivity in Cancer (GDSC) database. The statistical significance threshold was set at P < 0.001, and a random seed was set to 12345 to ensure the reproducibility of results. Pearson correlation analysis was performed to explore the correlation between CRRS and drug IC50, and drugs with |r| ≥ 0.5 were selected.

### 2.5 Construction and evaluation of nomogram

A nomogram was constructed based on CRRS and clinical variables (gender, age, tumor stage, T stage, N stage) to quantitatively predict 1-year, 3-year, and 5-year overall survival (OS) of patients:

Model construction: The “rms” package in R software was used to build a multivariate Cox regression model, which integrated CRRS and clinical variables to generate a nomogram. The “regplot” package was applied to optimize the visualization effect of the nomogram.

Model validation:

Time-dependent ROC curves were plotted using the “timeROC” package, and AUC values at different time points were calculated to evaluate the discriminative ability.

Calibration curves were generated using the “survival” package, and the Hosmer-Lemeshow test was performed to assess the consistency between predicted values and actual survival outcomes.

The C-index was calculated to quantify the overall predictive performance of the model, with a range of 0.5 to 1.0. A value closer to 1 indicates better predictive performance.

### 2.6 Rationale for cell line selection

COL11A1 gene expression profiles across multiple lung adenocarcinoma (LUAD) cell lines were retrieved from the Cancer Cell Line Encyclopedia (CCLE) database. Analysis results demonstrated that the A549 cell line exhibited substantially higher COL11A1 expression compared with other commonly used LUAD cell lines (**Supplementary Figure 1**), whereas the PC-9 cell line displayed low COL11A1 expression. Based on this baseline expression discrepancy, A549 and PC-9 were selected as model systems to ensure that subsequent functional assays could robustly validate the biological role of COL11A1.

### 2.7 Cell culture and lentiviral infection

The human lung adenocarcinoma cell lines A549 and PC-9 were purchased from the Cell Bank of Shanghai Institutes for Biological Sciences, Chinese Academy of Sciences. Cells were cultured in RPMI-1640 medium supplemented with 10% fetal bovine serum, 100 U/mL penicillin, and 100 μg/mL streptomycin. They were maintained in a cell incubator at 37 °C with 5% CO_2_ and saturated humidity, and the medium was replaced every 2–3 days.

Lentivirus and infection procedure: Lentiviruses for COL11A1 overexpression (OE-COL11A1), knockdown (sh-COL11A1, with 2 independent targeting sequences), and their corresponding negative controls (OE-NC, sh-NC) were purchased from Shanghai GeneChem Co., Ltd. Lentiviruses were added to cells in the logarithmic growth phase at a multiplicity of infection (MOI) of 10. Fresh medium was replaced 24 hours after infection. At 48 hours post-infection, 2 μg/mL puromycin was added for selection over 2 weeks to obtain stably expressing cell lines.

### 2.8 Colony formation assay

This assay was conducted to evaluate the long-term proliferation ability of cells, following the steps below:

Cell Seeding: Stably transfected cell lines were seeded into 6-well plates at a density of 500 cells per well, with 3 biological replicates set for each group.

Culture and Fixation: The plates were incubated in a 37 °C, 5% CO_2_ incubator for 14 days. After incubation, the culture medium was discarded, and the cells were fixed with pre-cooled methanol at room temperature for 15 minutes.

Staining and Quantification: The fixed cells were stained with 0.1% crystal violet (Sigma, Cat. No. C3886) at room temperature for 30 minutes. After thorough rinsing with running water, the plates were air-dried. Colonies with a diameter of ≥50 μm were counted using ImageJ software, and the colony formation rate was calculated using the formula:Colony formation rate = (Number of actual colonies/Number of seeded cells) × 100%.

### 2.9 Patients and specimens

A total of 52 pairs of matched lung adenocarcinoma (LUAD) tissues and adjacent normal tissues (located ≥ 5 cm away from the tumor margin) used in this study were obtained from the Department of Pathology, Shanxi Provincial People’s Hospital. All specimens were reconfirmed by two senior pathologists.

This study was approved by the Ethics Committee of Shanxi Provincial People’s Hospital (Approval No. 2021-196), and all procedures were strictly conducted in accordance with the Ethical Review Measures for Biomedical Research Involving Humans. Written informed consent was obtained from all patients or their legal guardians.

Tissue specimens were fixed with 4% paraformaldehyde, embedded in paraffin, and cut into 4 μm-thick serial sections for immunohistochemistry (IHC) experiments; some fresh tissues were stored at -80 °C for Western blot experiments.

### 2.10 Immunohistochemistry and immunofluorescence assays

#### 2.10.1 Immunohistochemistry staining

Tissue microarrays were constructed by selecting the core tumor regions and adjacent normal tissue regions from paraffin-embedded tissues. The staining procedure was as follows:

Tissue microarrays were constructed by punching core tissues from formalin-fixed paraffin-embedded (FFPE) tumor specimens and their matched adjacent normal tissue counterparts. For IHC staining, serial tissue sections were subjected to standard dewaxing and rehydration procedures prior to incubation with 3% hydrogen peroxide (H_2_O_2_) at room temperature (RT) for 10 min to quench endogenous peroxidase activity. Antigen retrieval was subsequently performed by heating the sections in citrate buffer (pH 6.0) for 15 min, and non-specific antibody binding was blocked by incubation with 5% bovine serum albumin (BSA) at RT for 30 min. The sections were then incubated with the corresponding primary antibodies at 4 °C overnight, followed by incubation with horseradish peroxidase (HRP)-conjugated secondary antibodies at RT for 30 min. Immunostaining was visualized using 3,3’-diaminobenzidine (DAB) as the chromogen, and the sections were counterstained with hematoxylin, dehydrated through a graded ethanol series, cleared in xylene, and mounted with neutral balsam.

All stained sections were scanned using a Panoramic MIDI scanner (3DHISTECH, Budapest, Hungary). The expression levels of target proteins were semi-quantitatively assessed by calculating the H-score with Panoramic Viewer v.1.15.3 software equipped with the Nuclear Quant plugin. The H-score was determined using the following formula: H-score = (percentage of weakly stained cells ×1) + (percentage of moderately stained cells ×2) + (percentage of strongly stained cells ×3), where the immunostaining intensity was categorized into three grades: 1 (weak), 2 (moderate), and 3 (strong) (19).

#### 2.10.2 Immunofluorescence staining

Stably transfected cell lines were seeded into laser confocal culture dishes, and the experiment was performed when the cell confluency reached 40%. The staining procedure was as follows:

1. Fixation with pre-cooled methanol at room temperature for 15 minutes;
2. Incubation with 0.3% Triton X-100 at room temperature for 10 minutes to increase cell membrane permeability;
3. Blocking with 5% BSA at room temperature for 30 minutes;
4. Incubation with primary antibody at 4 °C overnight;
5. Incubation with fluorescence-conjugated secondary antibody at room temperature for 1 hour in the dark;
6. Staining with 4’,6-diamidino-2-phenylindole (DAPI) at room temperature for 5 minutes in the dark;
7. After washing with phosphate-buffered saline (PBS) three times, images were observed and captured using a laser confocal microscope (Zeiss, LSM980).

### 2.11 Cell cycle assay

Cell lines were seeded into 6-well plates and starved in medium containing 1% fetal bovine serum (FBS) for 24 hours. Subsequently, the medium was replaced with complete medium, and the cells were cultured for an additional 24 hours.

Cells were harvested and processed using a Cell Cycle Detection Kit (Life-iLab, Cat. No. AC12L553). The cell cycle distribution was analyzed by flow cytometry.

### 2.12 Wound healing assay

Cell lines were seeded uniformly in 6-well plates and cultured at 37 °C with 5% CO_2_ until reaching 90%–100% confluency (monolayer formation).

Uniform wounds were scratched perpendicular to the well bottom on the cell monolayer using a sterile 100 μL pipette tip (2–3 parallel lines per well to avoid edge effects). Wells were gently washed three times with PBS to remove floating cells and scratch-induced debris, then refreshed with basic medium containing 1% FBS and returned to the incubator.

Wound areas were imaged under a microscope at 0 h, 24 h, and 48 h. Cell migration rate was calculated by measuring wound widths at different time points using the formula: Migration rate = [(Wound width at 0 h – Wound width at specific time point)/Wound width at 0 h] × 100%.

### 2.13 Transwell assay

Cell lines were resuspended in serum-free basal medium and adjusted to a concentration of 1×10^5^–5×10^5^ cells/mL. A 200 μL aliquot of this cell suspension was added to the upper chamber of Transwell inserts, while 800 μL of complete medium supplemented with 15% fetal bovine serum (FBS) was added to the lower chamber (air bubbles between the two chambers were carefully avoided).

Inserts were placed in 12-well plates and incubated at 37 °C with 5% CO_2_ for 24 h. After incubation, non-migrated cells in the upper chamber were gently removed using sterile cotton swabs. Migrated cells adhering to the bottom of inserts were fixed with 4% paraformaldehyde at room temperature for 20 min, stained with 0.1% crystal violet solution at room temperature for 15 min, and then gently washed three times with phosphate-buffered saline (PBS) to eliminate excess stain. Five random fields of view per insert were imaged under an inverted microscope, and the number of migrated cells in each field was counted to assess cell migration capacity.

### 2.14 qRT-PCR assay

Stably transfected cell lines were seeded in 6-well plates and cultured at 37 °C with 5% CO_2_. Cells were harvested when reaching 70%–80% confluency for subsequent experiments.

Total RNA was isolated using an RNA Extraction Kit (Beyotime, Cat. No. R0024) following the manufacturer’s protocol. RNA purity (A260/A280 ratio: 1.8–2.0) and concentration were quantified with a Nanodrop Ultra-Micro Spectrophotometer (Thermo Scientific, ND-2000) to confirm RNA integrity.

#### 2.14.1 cDNA reverse transcription

cDNA synthesis was performed using 1 μg of qualified total RNA as the template with a Reverse Transcription Kit (Beyotime, Cat. No. D7180S) on a PCR instrument. The reaction conditions were: 37 °C for 15 min (reverse transcription), 85 °C for 5 s (enzyme inactivation), and holding at 4 °C.

#### 2.14.2 qRT-PCR amplification

Synthesized cDNA was diluted 10-fold with RNase-free water and used for qRT-PCR. The 20 μL reaction system was prepared per the Quantitative Real-Time PCR Kit (Beyotime, Cat. No. D7601S) instructions, containing 10 μL 2×SYBR Green Mix, 0.4 μL forward primer, 0.4 μL reverse primer, 2 μL cDNA template, and 7.2 μL RNase-free water. Amplification was conducted on a real-time quantitative PCR instrument with the program:

- Pre-denaturation: 95 °C for 10 min;
- 40 cycles: 95 °C for 15 s (denaturation) and 60 °C for 1 min (annealing/extension);
- Melting curve analysis: 95 °C for 15 s, 60 °C for 1 min, 95 °C for 15 s (to verify amplification specificity).

#### 2.14.3 Data analysis

Glyceraldehyde-3-phosphate dehydrogenase (GAPDH) served as the internal reference gene. Relative expression of the target gene was calculated using the 2^(-ΔΔCt) method. Experiments were performed in triplicate, and statistical analyses were conducted to determine data differences.

### 2.15 ChIP assay

Stably transfected cell lines were seeded in 10 cm culture dishes and cultured at 37 °C with 5% CO_2_. At 80%–90% confluency, cells were cross-linked with 1% formaldehyde at room temperature for 10 min, and the reaction was terminated by adding glycine (final concentration: 0.125 M) for 5 min. After discarding the medium, cells were washed three times with pre-chilled PBS and harvested as pellets.

#### 2.15.1 Chromatin extraction and sonication

Cell pellets were lysed with nuclear lysis buffer (supplemented with protease inhibitors) by incubation on ice for 30 min. Nuclear lysates were sonicated using an ultrasonic disruptor (Diagenode, Bioruptor Pico) to fragment chromatin into 200–500 bp segments. After centrifugation at 12,000 rpm for 10 min at 4 °C, the supernatant (sonicated chromatin) was collected, and a small aliquot was used to verify fragment size.

#### 2.15.2 Immunoprecipitation

An aliquot of chromatin supernatant was incubated with 1–2 μg target protein-specific antibody (or IgG as negative control) on a slow shaker at 4 °C overnight. Protein A/G agarose beads (Beyotime, Cat. No. P2197S) were added the next day, and incubation continued at 4 °C for 2–4 h to form antibody-antigen-bead complexes. Complexes were collected by centrifugation at 3,000 rpm for 5 min at 4 °C, and pellets were sequentially washed three times with low-salt buffer, high-salt buffer, LiCl buffer, and TE buffer to remove non-specifically bound impurities.

#### 2.15.3 Cross-linking reversal and DNA purification

Elution buffer was added to washed pellets, and cross-linking was reversed by incubation at 65 °C for 4 h. Proteinase K was then added, followed by incubation at 55 °C for 1 h to degrade proteins. Immunoprecipitated DNA fragments were purified using a DNA Purification Kit (Beyotime, Cat. No. D0033).

#### 2.15.4 Detection and analysis

Purified DNA was used as a template for conventional PCR or qRT-PCR to detect the enrichment of target gene promoter regions (or specific binding sites). Enrichment folds were calculated to assess protein-DNA binding efficiency. Experiments were performed in triplicate to ensure result reliability.

### 2.16 EdU incorporation assay

Cells were seeded into laser confocal culture dishes, starved in medium containing 1% fetal bovine serum (FBS) for 24 hours, and then cultured in complete medium for another 24 hours. Cells were processed using the BeyoClick EdU-555 Kit (Beyotime, Cat. No. C0075L). Images were captured using a laser confocal microscope, and the proportion of EdU-positive cells was quantified.

### 2.17 CCK-8 assay

Cells were seeded into 96-well plates at a density of 1×10³ cells per well, with 5 technical replicates set for each group. The plates were incubated in a 37 °C incubator with 5% CO_2_, and cell viability was detected at 0, 24, 48, and 72 hours. At each time point, 10 μL of CCK-8 reagent (Life-iLab, Cat. No. AC11L054) was added to each well, followed by incubation at 37 °C for 1 hour. The absorbance (OD value) of cells at a wavelength of 450 nm was measured using a multi-function microplate reader (BioTek, Synergy HTX).

### 2.18 Treatment with PI3K inhibitor BYL719

BYL719 (NVP-BYL719), a highly selective PI3Kα inhibitor, was dissolved in dimethyl sulfoxide (DMSO) to prepare a 10 mM stock solution, which was stored at -20 °C in the dark. For *in vitro* assays, the stock solution was diluted to the desired working concentration with complete cell culture medium, with the final DMSO concentration in the medium strictly controlled to ≤ 0.1%.

The optimal administration dose of BYL719 was determined based on the findings of Fritsch et al. (20) and our preliminary experiments. As reported in the literature, BYL719 exerts a potent and specific inhibitory effect on the PI3K/AKT/mTOR signaling pathway in tumor cells within the concentration range of 10–20 μmol/L. To validate the optimal dose for our experimental setting, we treated COL11A1-overexpressing A549 and PC-9 cells with a gradient of BYL719 concentrations (0, 5, 10, 15, 20, and 25 μmol/L). Our results demonstrated that treatment with 20 μmol/L BYL719 significantly suppressed the phosphorylation levels of PI3K, AKT, and mTOR proteins. This concentration not only effectively reversed the malignant phenotypes of lung adenocarcinoma cells induced by COL11A1 overexpression, but also avoided overt cytotoxicity associated with higher doses. Thus, 20 μmol/L was designated as the optimal intervention concentration for subsequent functional rescue experiments.

Cells in the experimental group were incubated in complete medium supplemented with 20 μmol/L BYL719, whereas the control group was treated with an equal volume of medium containing 0.1% DMSO. Following continuous incubation for 24 h or 72 h, corresponding cell function assays were conducted.

### 2.19 Western blot assay

#### 2.19.1 Protein extraction

Stably transfected cells or frozen tissues were lysed in RIPA buffer supplemented with protease inhibitors (Roche, Cat. No. 04693159001) on ice for 30 min, then centrifuged at 12,000 rpm for 15 min at 4 °C. The supernatant was collected for subsequent use.

#### 2.19.2 Protein quantification

Protein concentration was determined using a BCA kit (Life-iLab, Cat. No. AP12L025). Sample loading volume was adjusted to load 30 μg protein per well.

#### 2.19.3 Electrophoresis and transfer

Proteins were separated by 10% SDS-PAGE, then transferred onto nitrocellulose (NC) membranes at 100 V constant voltage for 2 h. Membranes were blocked with 5% non-fat milk at room temperature for 1 h to block non-specific binding.

#### 2.19.4 Antibody incubation and development

Blocked membranes were incubated with primary antibodies at 4 °C overnight, washed three times with TBST, and then incubated with horseradish peroxidase (HRP)-conjugated secondary antibodies at room temperature for 1 h. Protein bands were visualized via enhanced chemiluminescence (ECL) kit.

### 2.20 Lung adenocarcinoma xenograft model

#### 2.20.1 Experimental animals

Six-week-old female BALB/c nude mice (Beijing Vital River Laboratory Animal Technology Co., Ltd.) were housed in a specific pathogen-free (SPF) facility under controlled conditions (22 ± 2 °C, 50 ± 10% humidity, 12-h light/dark cycle) and acclimated for 1 week prior to experimentation.

#### 2.20.2 Grouping and inoculation

Stably transfected A549 cells (OE-COL11A1, OE-NC, sh-COL11A1, sh-NC) were mixed 1:1 with Matrigel (Corning, Cat. No. 354234). Each mouse received a subcutaneous injection of 100 μL cell-Matrigel mixture (5×10^6^ cells) into the right dorsal region, with 5 mice per group.

#### 2.20.3 Tumor monitoring

From day 8 post-inoculation, tumor long (L) and short (W) diameters were measured every 3 days using a vernier caliper. Tumor volume was calculated as: Volume = L × W^2^/2.

#### 2.20.4 Euthanasia and specimen collection

On day 25 post-inoculation, mice were euthanized with a CO_2_ euthanasia device (Tianjin Hope Industry and Trade Co., Ltd., Model HOPE-MED8160) (100% CO_2_ at 20% container volume/min until respiratory arrest, plus 2 min extra exposure). Tumors were dissected, photographed, and weighed. Parts were fixed in 4% paraformaldehyde, and the rest stored at -80 °C for subsequent experiments.

#### 2.20.5 Ethical approval

The protocol was approved by the Ethics Committee of Shanxi Provincial People’s Hospital (No. 2021-191) and complied with the ARRIVE Guidelines.

### 2.21 Statistical analysis

All data were analyzed and visualized using R software (Version 4.1.0) with packages including ggplot2, survival, and survminer.

- Comparisons of differences between groups were performed using the Wilcoxon rank-sum test.
- Survival analysis was conducted using the Kaplan-Meier method to plot survival curves, and the log-rank test was used to compare survival differences between groups.

A P-value < 0.05 was considered statistically significant, with notations as follows: *P < 0.05, **P < 0.01, and*** P < 0.001.

## 3 Results

### 3.1 COL11A1 is highly expressed in LUAD and serves as an independent prognostic marker

**Figure 1:**
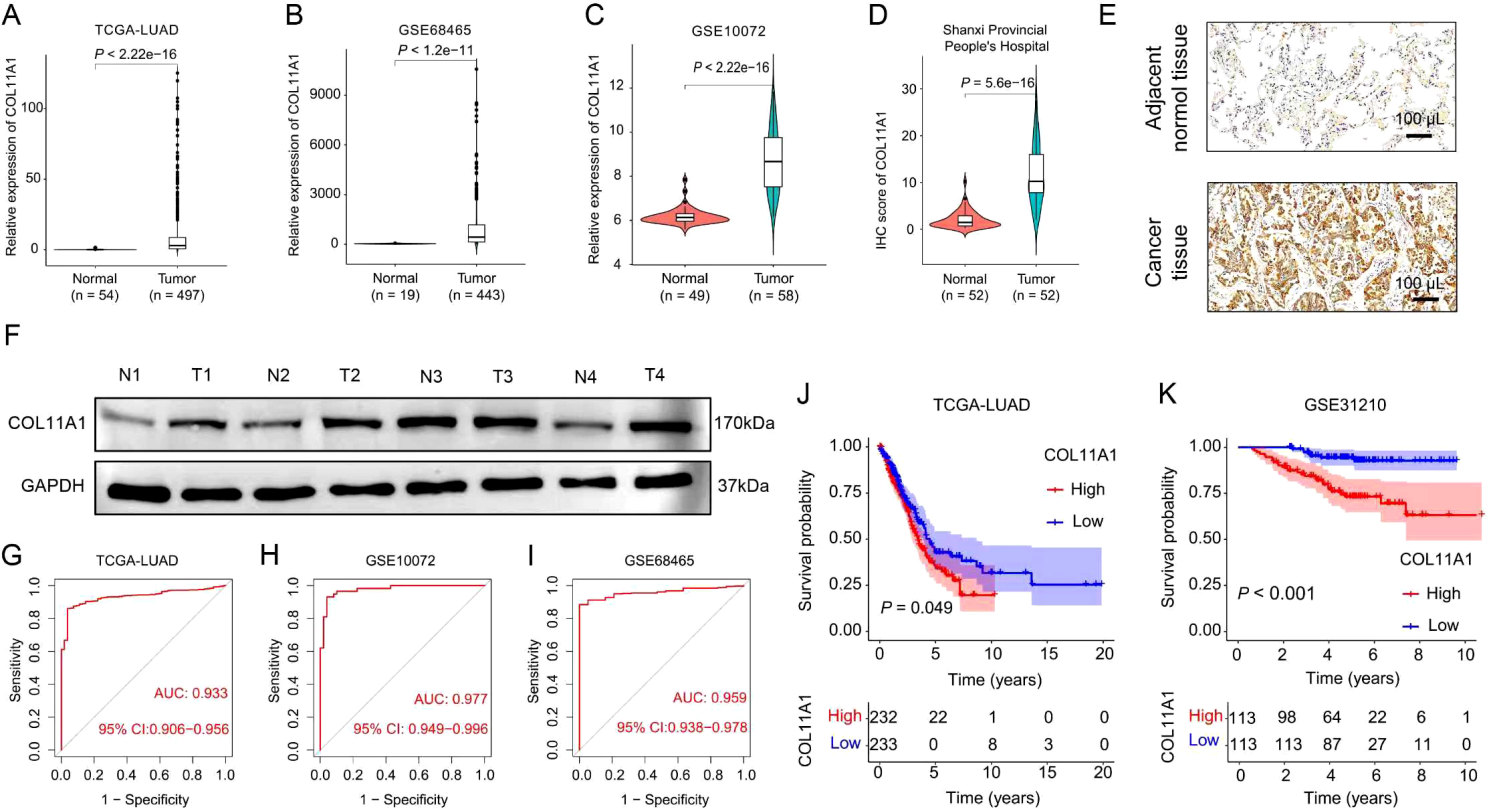
COL11A1 is highly expressed in lung adenocarcinoma (LUAD) and associated with poor prognosis. **(A–C)** Analysis of COL11A1 mRNA expression based on public datasets (TCGA-LUAD, GSE68465, GSE10072): The results showed that the mRNA level of COL11A1 in LUAD tissues was significantly higher than that in normal tissues (all P<0.001). **(D, E)** Immunohistochemistry (IHC) analysis of 52 paired LUAD tissues and adjacent normal tissues from Shanxi Provincial People’s Hospital: **(D)** Representative stained images; **(E)** Quantitative results of IHC scores showed that the protein expression of COL11A1 in tumor tissues was significantly higher than that in adjacent tissues (P<0.001). **(F)** Verification by Western blot assay: The protein level of COL11A1 in human LUAD tissues was significantly higher than that in adjacent normal tissues. **(G–I)** Diagnostic efficacy analysis of COL11A1: In the TCGA-LUAD, GSE10072, and GSE68465 datasets, the area under the receiver operating characteristic (ROC) curve (AUC) for distinguishing LUAD tissues from normal tissues based on COL11A1 expression was 0.933, 0.977, and 0.959, respectively. **(J, K)** Prognostic value analysis of COL11A1: **(J)** In the TCGA-LUAD cohort, the median overall survival (OS) of patients with high COL11A1 expression was significantly shorter than that of patients with low COL11A1 expression (27.7 months *vs*. 32.6 months, HR = 2.26, 95% CI: 1.76-2.90, P<0.001); **(K)** Consistent results were obtained in the GSE31210 validation cohort (52.2 months *vs*. 62.6 months, HR = 2.44, 95% CI: 1.58-3.78, P<0.001).

To clarify the expression characteristics of COL11A1 in lung adenocarcinoma (LUAD), this study analyzed three independent public datasets, namely TCGA-LUAD, GSE68465, and GSE10072. The results demonstrated that the mRNA level of COL11A1 was significantly upregulated in LUAD tissues compared with normal lung tissues (TCGA-LUAD: P<0.001; GSE68465: P<0.001; GSE10072: P<0.001; **Figures 1A–C**). The verification results of clinical samples were consistent with the above findings: Immunohistochemistry (IHC) analysis of 52 paired LUAD tissues and adjacent normal tissues collected from Shanxi Provincial People’s Hospital revealed that the protein expression level of COL11A1 in tumor tissues was significantly higher than that in adjacent tissues (**Figures 1D, E**). Western blot assay further confirmed the abnormally high expression of COL11A1 in LUAD tissues (**Figure 1F**).

Diagnostic efficacy analysis showed that in the TCGA-LUAD, GSE10072, and GSE68465 datasets, the area under the receiver operating characteristic (ROC) curve (AUC) of COL11A1 for distinguishing LUAD tissues from normal tissues was 0.933, 0.977, and 0.959, respectively (**Figures 1G–I**), indicating that COL11A1 has potent diagnostic value. Survival analysis indicated that in the TCGA-LUAD cohort, the overall survival (OS) of patients with high COL11A1 expression was significantly shorter than that of patients with low COL11A1 expression (median OS: 27.7 months *vs*. 32.6 months, HR = 2.26, 95% CI: 1.76-2.90, P<0.001; **Figure 1J**). Consistent results were obtained in the GSE31210 validation cohort (median OS: 52.2 months *vs*. 62.6 months, HR = 2.44, 95% CI: 1.58-3.78, P<0.001; **Figure 1K**).

### 3.2 COL11A1 promotes the proliferation and invasion of LUAD cells

**Figure 2:**
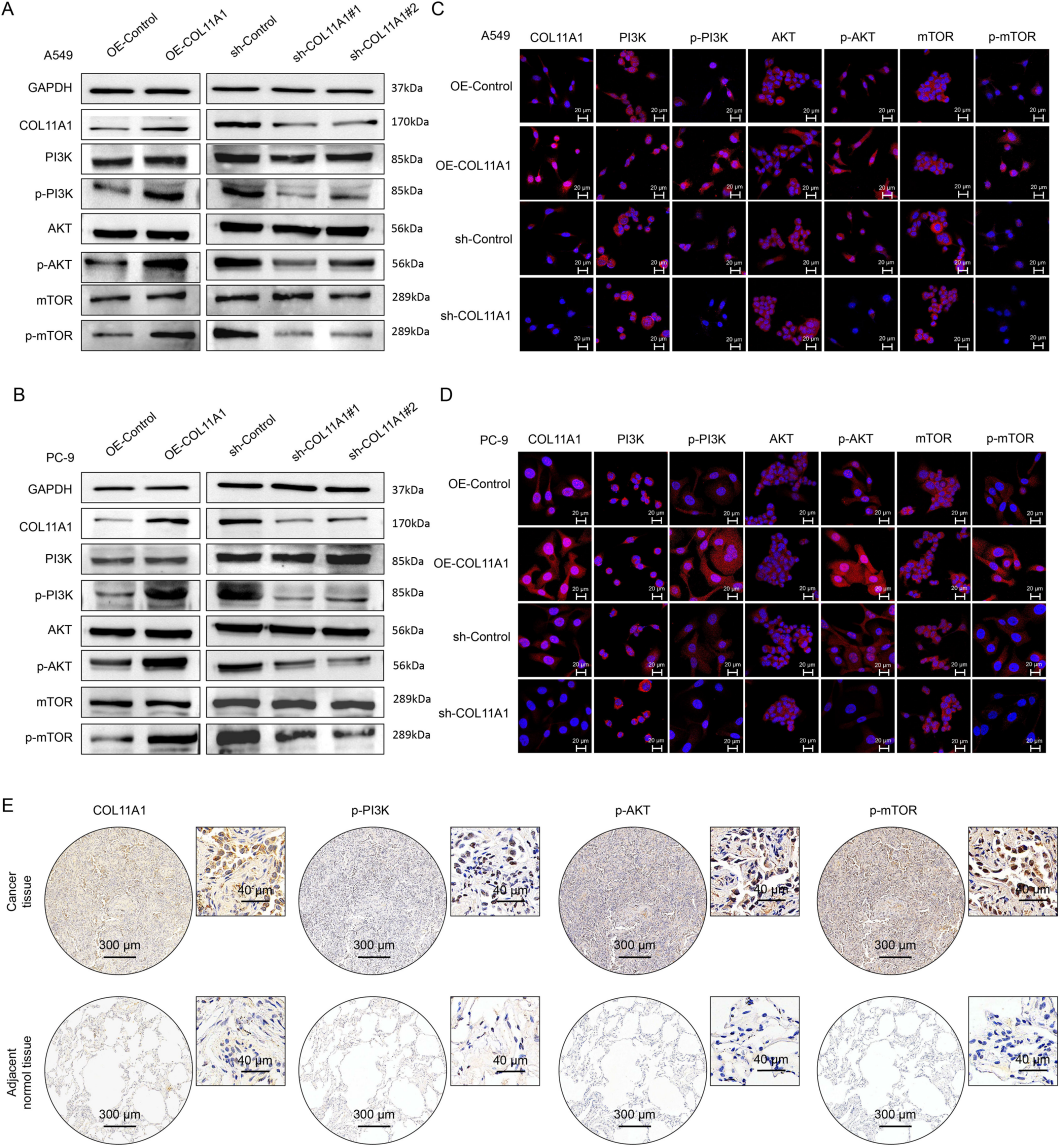
COL11A1 activates the PI3K/AKT/mTOR signaling pathway. **(A, B)** After overexpression/knockdown of COL11A1 in A549 and PC-9 cells, the expression levels of target proteins were detected by Western blot (WB) assay(n=3). **(C, D)** After overexpression/knockdown of COL11A1 in A549 and PC-9 cells, the expression levels of target proteins were detected by immunofluorescence assay(n=3). **(E)** Detection results of protein microarrays from clinical samples.

Validation of stable cell line construction: A549 and PC-9 cell lines with COL11A1 overexpression (OE-COL11A1), COL11A1 knockdown (sh-COL11A1, 2 independent targeting sequences), and their corresponding negative controls (OE-NC, sh-NC) were successfully constructed via lentiviral infection. Western blot analysis showed that the protein expression level of COL11A1 in the OE-COL11A1 group was higher than that in the OE-NC group, while the expression levels in the sh-COL11A1 groups (#1, #2) were significantly decreased (all P<0.001; **Figures 2A, B**). Immunofluorescence assay further verified the above regulatory effects (**Figures 2C, D**).

**Figure 3:**
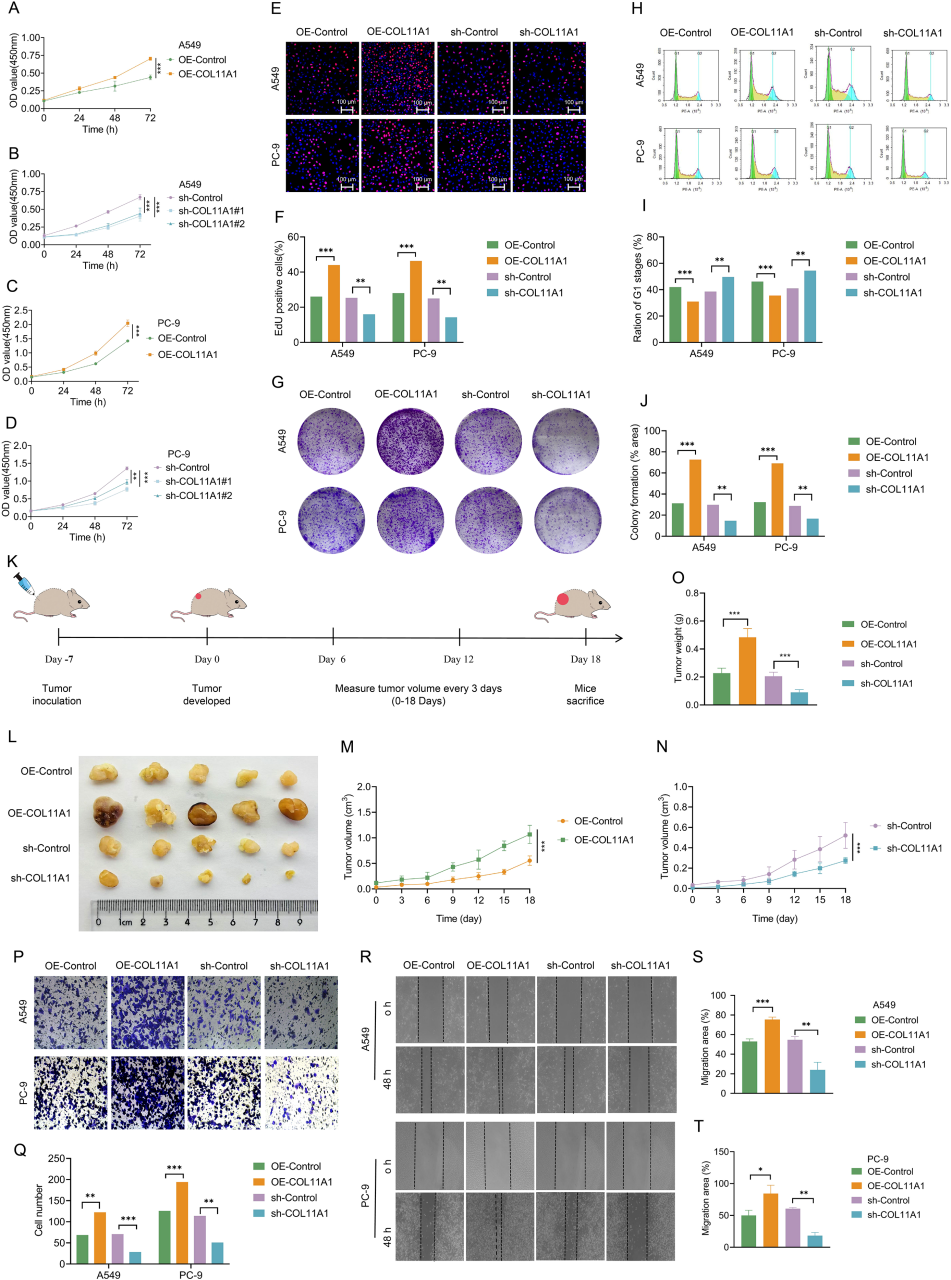
COL11A1 exerts pro-carcinogenic effects *in vitro* and *in vivo.***(A–D)** CCK-8 assay. COL11A1 overexpression significantly enhanced the proliferative capacity of A549 and PC-9 cells, while COL11A1 knockdown (using two independent short hairpin RNA sequences, shRNA) exerted the opposite effect (n=4 biological replicates). **(E, F)** EdU incorporation assay. COL11A1 overexpression significantly increased the percentage of EdU-positive cells in A549 and PC-9 cells, whereas COL11A1 knockdown led to the opposite trend. **(E)** Representative images; **(F)** Quantitative results (n=3 biological replicates). **(G, J)** Colony formation assay. COL11A1 overexpression promoted colony formation of A549 and PC-9 cells, while COL11A1 knockdown inhibited colony formation (n=3 biological replicates). **(G)** Representative images of colonies; **(J)** Quantitative results. **(H, I)** Flow cytometry analysis of cell cycle distribution. COL11A1 overexpression significantly reduced the proportion of G1-phase cells in A549 and PC-9 cells, while COL11A1 knockdown showed the opposite effect. **(H)** Representative flow cytometry plots; **(I)** Quantitative analysis (n=3 biological replicates). **(K)** Schematic diagram of the establishment of A549 cell-derived xenograft tumor model in BALB/c nude mice (subcutaneous inoculation; n=5 mice per group). **(L)** On day 25 after A549 cell inoculation, mice were euthanized, and tumor tissues were collected and photographed. **(M, N)** Tumor size in mice was measured starting from day 7 after A549 cell inoculation, with measurements taken every 3 days, and tumor growth curves were plotted. **(O)** At the end of the experiment, tumor tissues were collected and weighed. **(P, Q)** Transwell assay. COL11A1 overexpression promoted the migration of A549 and PC-9 cells, while COL11A1 knockdown inhibited migration (n=3 biological replicates). **(P)** Representative images; **(Q)** Quantitative results. **(R–T)** Wound healing assay. COL11A1 overexpression promoted the migration of A549 and PC-9 cells, while COL11A1 knockdown inhibited migration (n=3 biological replicates). **(R)** Representative images; **(S, T)** Quantitative results. Experiments were performed using sh-COL11A1#1. *P<0.05; **P<0.01; ***P<0.001.

Results of *in vitro* functional experiments. Cell proliferation ability:CCK-8 assay showed that after 72 hours of culture, the OD450 values of A549 and PC-9 cells in the OE-COL11A1 group were significantly higher than those in the OE-NC group (A549: 0.70 ± 0.02 *vs* 0.44 ± 0.03, P<0.001; PC-9: 1.98 ± 0.12 *vs* 1.42 ± 0.04, P<0.001; **Figures 3A–C**). In contrast, the OD450 values in the sh-COL11A1 group were significantly decreased (A549 sh-#1: 0.40 ± 0.07 *vs* 0.67 ± 0.04, P<0.001; PC-9 sh-#1: 0.77 ± 0.05 *vs* 1.36 ± 0.04, P<0.001; **Figures 3B–D**).EdU staining assay confirmed that the proportion of EdU-positive cells in the OE-COL11A1 group was significantly increased (A549: 44.0 ± 4.6% *vs* 26.0 ± 2.7%, P<0.001; PC-9: 46.3 ± 4.0% *vs* 28.0 ± 3.6%, P<0.001), while it was significantly decreased in the sh-COL11A1 group (A549: 16.0 ± 4.2% *vs* 25.3 ± 4.0%, P<0.01; PC-9: 14.3 ± 1.6% *vs* 25.0 ± 4.0%, P<0.01; **Figures 3E, F**).Colony formation assay revealed that the colony formation rate in the OE-COL11A1 group was significantly higher than that in the control group (A549: 72.7 ± 1.7% *vs* 31.2 ± 0.4%, P<0.001; PC-9: 69.1 ± 0.6% *vs* 32.4 ± 0.6%, P<0.001), whereas it was significantly reduced in the sh-COL11A1 group (A549: 14.7 ± 0.4% *vs* 29.8 ± 0.35%, P<0.01; PC-9: 16.6 ± 0.56% *vs* 28.7 ± 0.45%, P<0.01) (**Figures 3G–J**).

Cell cycle distribution: Flow cytometry analysis showed that the proportion of cells in the G0/G1 phase was significantly decreased in the OE-COL11A1 group (A549: 30.9 ± 0.8% *vs* 41.9 ± 2.3%, P<0.001; PC-9: 35.6 ± 1.4% *vs* 46.1 ± 0.4%, P<0.001), while the sh-COL11A1 group exhibited the opposite trend (A549: 49.7 ± 1.2% *vs* 38.6 ± 1.5%, P<0.01; PC-9: 54.5 ± 1.2% *vs* 41.0 ± 1.8%, P<0.01) (**Figures 3H, I**).

Cell migration and invasion: Transwell assay demonstrated that the number of transmembrane cells in the OE-COL11A1 group was significantly increased (A549: 122.6 ± 10.5 cells *vs* 68.7 ± 2.5 cells, P<0.01; PC-9: 194.3 ± 6.1 cells *vs* 126.0 ± 5.0 cells, P<0.001), while it was significantly reduced in the sh-COL11A1 group (A549: 28.3 ± 1.5 cells *vs* 70.7 ± 6.0 cells, P<0.001; PC-9: 51.0 ± 2.7 cells *vs* 114.0 ± 14.0 cells, P<0.01; **Figures 3P, Q**). Wound healing assay showed that after 48 hours, the wound healing rate in the OE-COL11A1 group was significantly higher than that in the control group (A549: 75% *vs* 46%, P<0.001; PC-9: 80% *vs* 61%, P<0.05), while it was significantly decreased in the sh-COL11A1 group (A549: 24% *vs* 55%, P<0.01; PC-9: 18% *vs* 61%, P<0.01; **Figures 3R–T**).

Results of *in vivo* functional experiments: In the nude mouse model with A549 cell xenografts, on the 25th day after inoculation, both the tumor volume (1.07 ± 0.18 cm³ *vs* 0.52 ± 0.07 cm³, P<0.001) and weight (0.48 ± 0.06 g *vs* 0.23 ± 0.04 g, P<0.001) in the OE-COL11A1 group were significantly larger than those in the OE-NC group. In contrast, the tumor volume (0.27 ± 0.03 cm³ *vs* 0.52 ± 0.13 cm³, P<0.01) and weight (0.09 ± 0.02 g *vs* 0.21 ± 0.03 g, P<0.01) in the sh-COL11A1 group were significantly smaller than those in the sh-NC group (**Figures 3L–O**).

In summary, based on the successful construction of stable LUAD cell lines with regulated COL11A1 expression, this study confirmed through *in vitro* functional experiments that COL11A1 could significantly promote the proliferation ability, cell cycle progression, migration and invasion capabilities of A549 and PC-9 cells. Furthermore, *in vivo* xenotransplantation experiments further verified that COL11A1 could significantly accelerate the growth of LUAD tumors. Collectively, these findings clearly demonstrate the promotional effect of COL11A1 on the proliferation and invasion of LUAD cells.

### 3.3 COL11A1 exerts a pro-carcinogenic effect by activating the PI3K/AKT/mTOR pathway

The PI3K-AKT-mTOR signaling pathway is a key intracellular pathway for transmitting growth signals. Its abnormal activation is involved in almost the entire process of malignant tumor progression, providing “driving force” for malignant behaviors of tumor cells such as unregulated growth, invasion and metastasis (21). Western blot results showed that after overexpression of COL11A1 in A549 and PC-9 cells, the phosphorylation levels of PI3K, AKT and mTOR were significantly increased, while the opposite trend was observed after COL11A1 knockdown (**Figures 3A, B**). Immunofluorescence experiments further confirmed this result (**Figures 3C, D**). IHC detection of tissue microarrays from clinical samples showed that the expression level of COL11A1 in LUAD tissues exhibited the same changing trend as that of p-PI3K, p-AKT and p-mTOR (**Figure 3E**).

**Figure 4:**
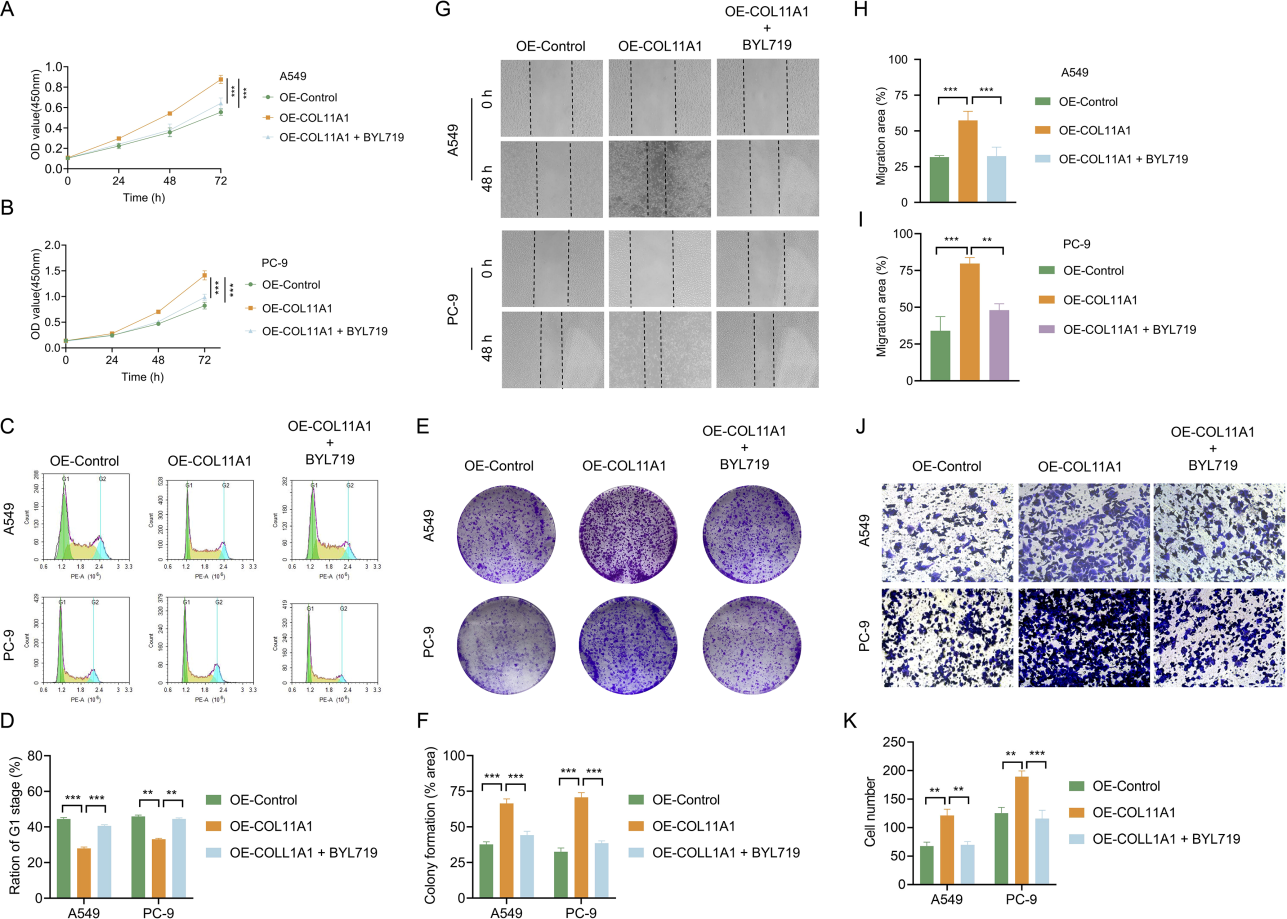
PI3K inhibitor BYL719 reverses the malignant phenotype of LUAD cells mediated by COL11A1. **(A, B)** CCK-8 proliferation assay: After 72 hours of culture, the OD450 values of A549 and PC-9 cells in the OE-COL11A1 group were significantly higher than those in the OE-NC group (A549: 0.88 ± 0.04 *vs* 0.56 ± 0.03, PC-9: 1.41 ± 0.09 *vs* 0.82 ± 0.07, all P<0.001); after adding 20 μM PI3K inhibitor BYL719, the OD450 values of the OE-COL11A1+BYL719 group significantly decreased to the control level (A549: 0.64 ± 0.05, PC-9: 0.99 ± 0.06, all P<0.001, n=5). **(C, D)** Cell cycle analysis: The proportion of cells in the G0/G1 phase in the OE-COL11A1 group was significantly reduced (A549: 28.0 ± 0.7% *vs* 44.4 ± 0.9%, PC-9: 33.1 ± 0.5% *vs* 46.1 ± 0.8%, all P<0.01); after treatment with BYL719 (20 μM, 24 h), the proportion of G0/G1 phase cells increased (A549: 40.5 ± 0.7%, PC-9: 44.5 ± 0.7%, all P<0.001, n=3). **(E, F)** Colony formation assay: **(E)** Representative images; **(F)** Quantitative results showed that the colony formation rate of the OE-COL11A1 group was significantly increased (A549: 66.4 ± 3.2% *vs* 39.3 ± 0.6%, PC-9: 70.7 ± 3.3% *vs* 32.2 ± 0.07%, P<0.001), and significantly decreased to (A549: 44.2 ± 2.7%, PC-9: 38.5 ± 1.7%, P<0.001, n=3) after adding BYL719. **(G, H)** Wound healing assay: **(G)** Representative images (scale bar = 200 μm); **(H, I)** Quantitative results showed that the 48-hour wound healing rate of the OE-COL11A1 group was significantly increased (A549: 57.3 ± 6.1% *vs* 31.7 ± 1.2%, PC-9: 79.7 ± 4.2% *vs* 34.0 ± 9.6%, P<0.001), and decreased to (A549: 32.3 ± 6.0%, PC-9: 48.4 ± 4.4%, P<0.001, n=3) after adding BYL719. **(J, K)** Transwell invasion assay: **(J)** Representative images (scale bar = 100 μm); **(K)** Quantitative results showed that the number of transmembrane cells in the OE-COL11A1 group was significantly increased (A549: 121.0 ± 1.5 *vs* 67.3 ± 7.1, PC-9: 189.3 ± 2.5 *vs* 125.7 ± 9.7, P<0.001), and decreased to (A549: 69.7 ± 5.5, PC-9: 116.0 ± 3.6, P<0.001, n=3) after adding BYL719. *P<0.05, **P<0.01, ***P<0.001.

To confirm that COL11A1 promotes the proliferation and metastasis of LUAD cells by activating the PI3K-AKT-mTOR signaling pathway, we treated COL11A1-overexpressing (OE-COL11A1) cell lines (A549 and PC-9) with the PI3K inhibitor BYL719 (20 μmol) and detected cell proliferation using the CCK-8 assay. The results showed that after 72 hours of culture, the OD value of the OE-COL11A1 + BYL719 group was significantly lower than that of the OE-COL11A1 group (A549: 0.64 ± 0.05 *vs* 0.88 ± 0.04, P<0.001; PC-9: 0.99 ± 0.06 *vs* 1.41 ± 0.09, P<0.001; **Figures 4A, B**). Cell cycle analysis revealed that the proportion of cells in the G0/G1 phase in the OE-COL11A1 + BYL719 group (20 μmol, treated for 24 h) was significantly higher than that in the OE-COL11A1 group (A549: 40.5 ± 0.7% *vs* 28.0 ± 0.7%, P<0.001; PC-9: 44.5 ± 0.7% *vs* 33.1 ± 0.5%, P<0.001; **Figures 4C, D**). Results of the colony formation assay indicated that BYL719 (10 μmol) could effectively reverse COL11A1 overexpression-mediated cell proliferation (colony formation rate: OE-COL11A1 + BYL719 *vs* OE-COL11A1; A549: 44.2 ± 2.7% *vs* 66.4 ± 3.2%, P<0.001; PC-9: 38.5 ± 1.7% *vs* 70.7 ± 3.3%, P<0.001; **Figures 4E, F**).The wound healing assay (cell migration rate: OE-COL11A1 + BYL719 *vs* OE-COL11A1; A549: 32.3 ± 6.0% *vs* 57.3 ± 6.1%, P<0.001; PC-9: 48.4 ± 4.4% *vs* 79.7 ± 4.2%, P<0.001; **Figures 4G–I**) and Transwell assay (number of invasive cells: OE-COL11A1 + BYL719 *vs* OE-COL11A1; A549: 69.7 ± 5.5 *vs* 121.0 ± 1.5, P<0.01; PC-9: 116.0 ± 3.6 *vs* 189.3 ± 2.5, P<0.001) also confirmed that BYL719 could effectively inhibit COL11A1 overexpression-mediated cell migration and invasion.

In conclusion, through WB, immunofluorescence, and clinical tissue microarray IHC detection, this study confirmed that COL11A1 could significantly upregulate the phosphorylation levels of key molecules in the PI3K/AKT/mTOR pathway in LUAD. Further intervention experiments using the PI3K inhibitor BYL719 showed that this inhibitor could effectively reverse the enhancement of LUAD cell proliferation, cycle progression, migration, and invasion mediated by COL11A1 overexpression. These findings clearly demonstrate that COL11A1 exerts a pro-carcinogenic effect by activating the PI3K/AKT/mTOR pathway.

### 3.4 Transcription factor TWIST directly binds to the COL11A1 promoter and positively regulates its expression

**Figure 5:**
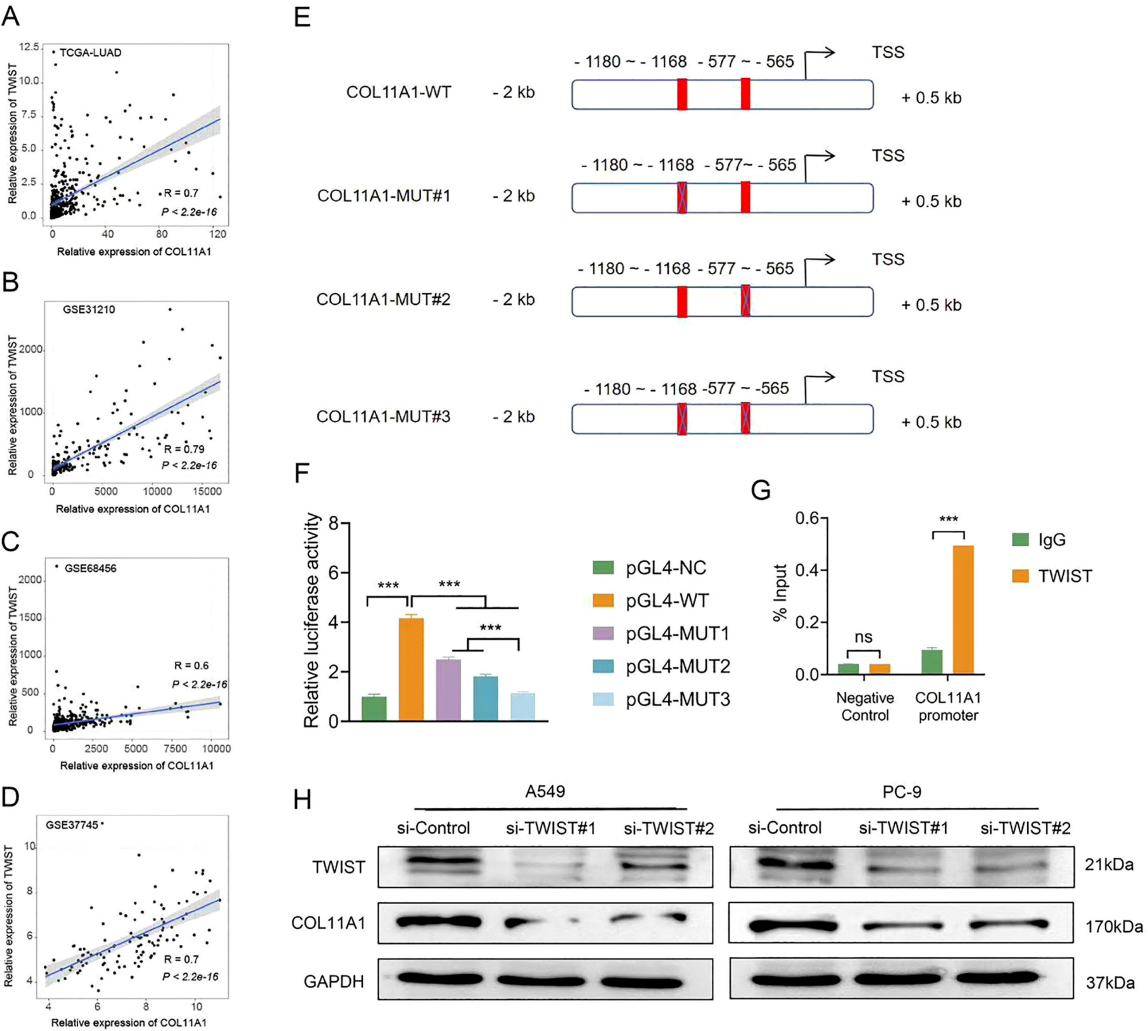
Transcription factor TWIST directly binds to the COL11A1 promoter and positively regulates its expression. **(A–D)** Co-expression analysis: In the TCGA-LUAD, GSE31210, GSE37745, and GSE68465 datasets, the mRNA levels of TWIST and COL11A1 showed a significant positive correlation (Pearson correlation coefficients r = 0.70, 0.79, 0.60, 0.70, respectively; all P<0.001). **(E)** Prediction via JASPAR database: There are 2 potential TWIST binding sites in the COL11A1 promoter region (-1500~+500 bp), namely the distal site (-1180~-1168 bp) and the proximal site (-577~-565 bp). **(F)** Dual-luciferase reporter gene assay: After co-transfecting TWIST expression vector and wild-type (WT) COL11A1 promoter vector into PC-9 cells, the luciferase activity increased by 3.17 folds (P<0.001); mutation of the distal/proximal site alone reduced the activity by 40%/56%, respectively; mutation of both sites reduced the activity by 73% (all P<0.001, n=3). **(G)** Chromatin Immunoprecipitation (ChIP) assay: In A549 cells, the enrichment fold of the TWIST antibody for the proximal region (-577~-565 bp) of the COL11A1 promoter was 5.44 folds, which was significantly higher than that of the IgG control (P<0.001, n=3). **(H)** Western blot verification: After siRNA-mediated TWIST knockdown, the protein level of COL11A1 in A549 and PC-9 cells was significantly decreased (n=3). ***P<0.001.

To explore the regulatory mechanism underlying COL11A1 expression in LUAD, we used public datasets to identify transcription factors co-expressed with COL11A1. The results showed that in the TCGA-LUAD, GSE31210, GSE68465, and GSE37745 datasets, the mRNA level of the transcription factor TWIST was significantly positively correlated with that of COL11A1 (r=0.7, 0.79, 0.6, 0.7; all P<0.001; **Figures 5A–D**). Prediction via the JASPAR database revealed that there are 2 potential TWIST binding sites in the COL11A1 promoter region (-1500~+500 bp): a distal site (-1180~-1168 bp) and a proximal site (-577~-565 bp; **Figure 5E**). Dual-luciferase reporter gene assay showed that after co-transfection with the TWIST expression vector, the luciferase activity of the wild-type COL11A1 promoter increased by 3.17 folds (P<0.001); after double-site mutation, the activity decreased by 73% (P<0.001); when the distal or proximal site was mutated alone, the activity decreased by 40% and 56%, respectively (all P<0.001; **Figure 5F**), suggesting that the proximal site exerts a stronger regulatory effect. ChIP assay confirmed that TWIST could directly bind to the proximal region (-577~-565 bp) of the COL11A1 promoter, with an enrichment fold of 5.44 (P<0.001; **Figure 5G**). Western blot analysis showed that after siRNA-mediated TWIST knockdown, the protein expression level of COL11A1 in A549 and PC-9 cells was significantly decreased (all P<0.001; **Figure 5H**).

In conclusion, through the analysis of multiple public LUAD datasets, this study found a significant positive correlation between the mRNA levels of transcription factor TWIST and COL11A1. The JASPAR database predicted potential TWIST binding sites in the COL11A1 promoter region. Further dual-luciferase reporter gene assay confirmed that TWIST could activate the COL11A1 promoter activity, with the proximal binding site showing a stronger regulatory effect. ChIP assay clearly demonstrated that TWIST could directly bind to the proximal region of the COL11A1 promoter. Meanwhile, TWIST knockdown could significantly reduce the COL11A1 protein expression in LUAD cells. These findings fully confirm that transcription factor TWIST can directly bind to the COL11A1 promoter and positively regulate its expression.

### 3.5 Construction and validation of COL11A1-related risk score and nomogram

#### 3.5.1 CRRS construction process

**Figure 6:**
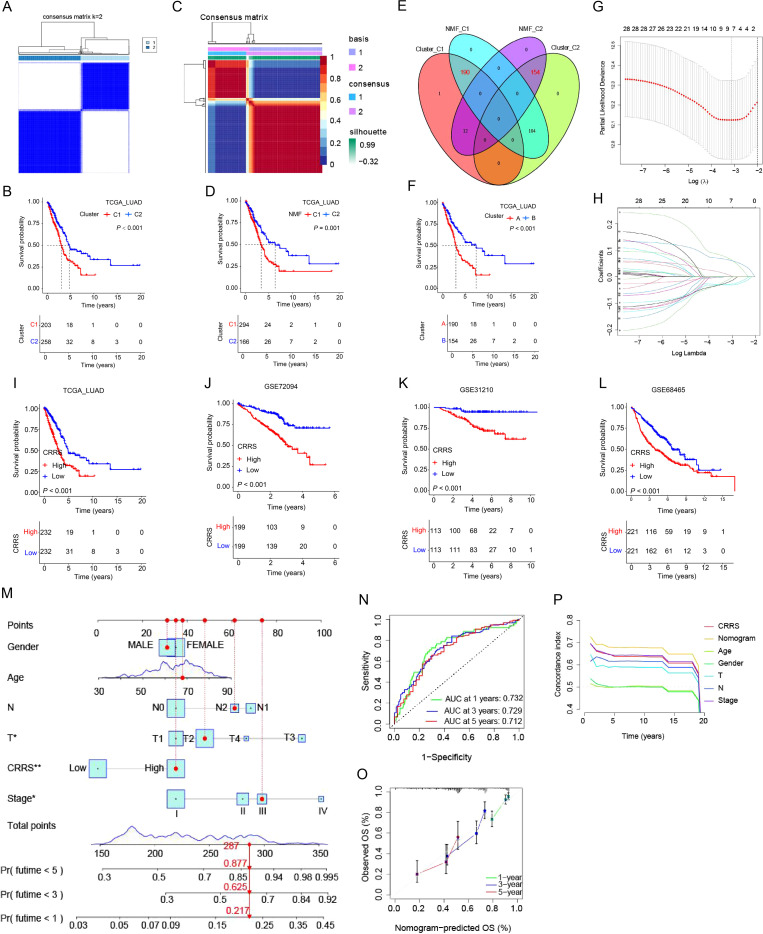
Construction and validation of COL11A1-related risk score (CRRS) and nomogram. **(A)** Consensus Clustering (CC) analysis: In the TCGA-LUAD cohort, patients were divided into 2 subgroups (C1, C2) based on COL11A1-coexpressed genes. The consensus matrix heatmap indicated good clustering stability (k=2). **(B)** Survival analysis of CC subgroups: The median overall survival (OS) of patients in subgroup C2 was significantly longer than that in C1 (33.2 months *vs* 26.8 months, P<0.05). **(C)** Non-negative Matrix Factorization (NMF) clustering: In the TCGA-LUAD cohort, clustering stability was optimal when rank=2 (cophenetic coefficient = 0.92), and patients were divided into 2 subgroups (N1, N2). **(D)** Survival analysis of NMF subgroups: The median OS of patients in subgroup N2 was significantly longer than that in N1 (36.1 months *vs* 27.2 months, P<0.01). **(E)** Venn diagram: The intersection of subgroup C2 (from CC clustering) and subgroup N2 (from NMF clustering) was defined as the “low-risk subgroup (cluster B)”, and the remaining patients were classified as the “high-risk subgroup (cluster A)”. **(F)** Survival analysis of cluster subgroups: The median OS of patients in cluster B was significantly longer than that in cluster A (36.6 months *vs* 26.8 months, P<0.05). **(G, H)** LASSO-Cox regression analysis: **(G)** λ value selection curve (10-fold cross-validation); **(H)** Coefficient path diagram. Finally, 7 core genes (LOXL2, CYP4B1, CA4, SFTPB, CRTAC1, TNNT1, KRT6A) were screened to construct CRRS, with the formula:score = 0.0729×LOXL2 - 0.0307×CYP4B1 - 0.0143×CA4 - 0.0294×SFTPB - 0.0227×CRTAC1 + 0.0099×TNNT1 + 0.0650×KRT6A. **(I-L)** Prognostic validation of CRRS: Patients were divided into high-/low-risk groups according to the median CRRS. In **(I)** TCGA-LUAD training set (25.3 months *vs* 38.6 months, HR = 3.08, P<0.001), **(J)** GSE31210 (45.8 months *vs* 68.3 months, HR = 5.17, P<0.001), **(K)** GSE68465 (38.5 months *vs* 65.2 months, HR = 6.87, P<0.001), and **(L)** GSE72094 (32.8 months *vs* 59.4 months, HR = 5.95, P<0.001) validation cohorts, the median OS of the high-risk group was significantly shortened. **(M)** Nomogram construction: CRRS was integrated with clinical variables (age, gender, tumor stage, T stage, N stage) to generate a nomogram for predicting 1-year, 3-year, and 5-year OS of LUAD patients. **(N)** Discrimination evaluation of the nomogram: Time-dependent ROC curve showed that the area under the curve (AUC) of the nomogram for predicting 1-year, 3-year, and 5-year OS was 0.73, 0.73, and 0.71, respectively (all P<0.001). **(O, P)** Accuracy evaluation of the nomogram: **(O)** Calibration curve showed high consistency between the OS predicted by the nomogram and the actual OS; **(P)** C-index comparison showed that the C-index of the nomogram was superior to that of other individual indicators.

With TCGA-LUAD as the training set and GSE68465, GSE72094, and GSE31210 as validation sets, the CRRS was constructed as follows: First, 182 COL11A1-coexpressed genes were screened (correlation coefficient ≥0.5, P ≤ 0.001; **Supplementary Table 1**). Based on the expression profiles of these genes, consensus clustering (CC) and non-negative matrix factorization (NMF) were used to stratify patients in the TCGA-LUAD cohort: For CC analysis, after evaluating K values from 2 to 10, the optimal K was determined as 2, dividing patients into Cluster C1 (203 cases) and Cluster C2 (258 cases); the median overall survival (OS) of Cluster C2 was significantly longer (33.2 months *vs* 26.8 months, P<0.05; **Figures 6A, B**). For NMF analysis, after assessing ranks from 2 to 10, the optimal rank was set as 2, classifying patients into Cluster N1 and Cluster N2 (166 cases in Cluster N2); the median OS of Cluster N2 was significantly longer (36.1 months *vs* 27.2 months, P<0.01; **Figures 6C, D**).The intersection of the two clustering results was used to further categorize patients into Cluster A (190 cases, high-risk) and Cluster B (154 cases, low-risk; **Figure 6E**), with the median OS of Cluster B being significantly longer than that of Cluster A (36.6 months *vs* 26.8 months, P<0.05; **Figure 6F**). Using Clusters A and B as the grouping standard, 81 prognosis-related differentially expressed genes (DEGs) were screened (|logFC|≥2, adjusted P ≤ 0.01; **Supplementary Table 2**). LASSO-Cox regression analysis was then performed to select 7 core genes (including LOXL2 and CYP4B1), and the CRRS was constructed with the formula:score = 0.0729×LOXL2 - 0.0307×CYP4B1 - 0.0143×CA4 - 0.0294×SFTPB - 0.0227×CRTAC1 + 0.0099×TNNT1 + 0.0650×KRT6A (**Figures 6G, H**).

#### 3.5.2 Prognostic evaluation efficacy of CRRS

Patients in the training and validation sets were divided into high-risk and low-risk groups based on the median CRRS:

Training set (TCGA-LUAD): The median OS of the high-risk group was significantly shorter than that of the low-risk group (25.3 months *vs* 38.6 months, HR = 3.08, 95% CI: 2.35-4.03, P<0.001; **Figure 6I**); Validation sets (GSE31210, GSE68465, GSE72094): The median OS of the high-risk group was significantly shortened in all validation sets (45.8 months *vs* 68.3 months, 38.5 months *vs* 65.2 months, 32.8 months *vs* 59.4 months, all P<0.001; **Figures 6J–L**). Drug sensitivity analysis revealed that CRRS was significantly negatively correlated with the half-maximal inhibitory concentration (IC50) of 11 chemotherapeutic drugs (|r|≥0.5, all P<0.001), including BI-2536 (r=-0.54), epothilone B (r=-0.58), and A770041 (r=-0.52; **Supplementary Figure 2**). This suggests that patients with high CRRS may be more sensitive to these drugs. In addition, a nomogram was constructed by integrating CRRS with clinical variables (age, gender, tumor stage, T stage, N stage; **Figure 6M**). ROC analysis showed that the area under the curve (AUC) for predicting 1-year, 3-year, and 5-year OS was all >0.71 (**Figure 6N**). The calibration curve demonstrated a high consistency between the predicted and actual outcomes (**Figure 6O**). The C-index indicated that the performance of this nomogram was superior to that of other individual indicators (**Figure 6P**).

In conclusion, CRRS can not only effectively distinguish the prognostic risk of LUAD patients (with significantly shortened median OS in the high-risk group) but also provide certain guidance for medication selection. Furthermore, the nomogram constructed by integrating CRRS with clinical variables exhibits better predictive performance than single indicators, offering a reliable quantitative tool for the prognostic evaluation of LUAD patients.

## 4 Discussion

As the most prevalent subtype of lung cancer, lung adenocarcinoma (LUAD) has a 5-year survival rate of less than 20%, which highlights the urgent need to identify novel molecular targets and prognostic tools (22). This study systematically investigated the role of COL11A1 in LUAD and identified three core findings: first, COL11A1 is specifically overexpressed in LUAD and associated with poor prognosis, exhibiting potential as a diagnostic and prognostic marker; second, this study is the first to reveal the molecular axis by which “the transcription factor TWIST directly regulates COL11A1 expression and drives the malignant progression of LUAD via activating the PI3K/AKT/mTOR pathway”; third, the COL11A1-related risk score (CRRS) constructed based on COL11A1-coexpressed genes and the nomogram integrated with clinical variables provide practical tools for prognostic evaluation and precision medication of LUAD. These findings supplement the research on the regulatory mechanism of COL11A1 expression in LUAD and its clinical translation, and also offer new theoretical basis and technical support for the precise diagnosis and treatment of LUAD.

### 4.1 Expression characteristics and prognostic significance of COL11A1 in LUAD

Through the validation of multiple datasets (TCGA-LUAD, GSE series) and 52 clinical specimens, this study confirmed that both the mRNA and protein levels of COL11A1 were significantly upregulated in LUAD tissues, and the median overall survival (OS) of patients with high COL11A1 expression was significantly shortened (TCGA-LUAD: 27.7 months *vs*. 32.6 months, P<0.001). More importantly, the area under the receiver operating characteristic (ROC) curve (AUC) of COL11A1 for distinguishing LUAD tissues from normal tissues was all >0.93, indicating a diagnostic efficacy superior to that of most reported LUAD markers [e.g., the AUC of ARlncRNAs is approximately 0.72 (23)]. This suggests that COL11A1 may serve as a potential indicator for the early diagnosis of LUAD. This result is consistent with the report by Shen et al. (24) that “COL11A1 is overexpressed in non-small cell lung cancer (NSCLC) and promotes cell proliferation”. However, this study further focuses on the LUAD subtype and confirms the specific expression pattern of COL11A1 in LUAD through multi-technical validation (e.g., immunohistochemistry [IHC], Western blot), laying a foundation for subsequent mechanistic research.

Furthermore, the pro-carcinogenic role of COL11A1 in other malignant tumors has been widely reported (e.g., high COL11A1 expression is associated with tamoxifen resistance in breast cancer (25), and COL11A1 drives invasion in ovarian cancer (26)). In this study, we systematically confirmed its regulatory effects on cell cycle, migration, and invasion in LUAD: *In vitro* experiments showed that COL11A1 overexpression reduced the proportion of A549 and PC-9 cells in the G1 phase by 26.3% and 22.8%, respectively, and increased the number of Transwell transmembrane cells by 78.5% and 54.2% (both P<0.001); *In vivo* xenotransplantation models further verified that COL11A1 could increase tumor volume by 105.8% (P<0.001). These results suggest that COL11A1 may exert its effects through a conserved mechanism of “promoting proliferation and invasion” across different cancers. However, its tissue-specific regulatory network still requires further exploration.

### 4.2 The TWIST-COL11A1-PI3K/AKT/mTOR axis

Exploring the key regulatory mechanisms underlying LUAD progression constitutes the core innovation of this study. First, through co-expression analysis across multiple datasets and ChIP experiments, we identified for the first time that the transcription factor TWIST can directly bind to the proximal region (-577~-565 bp) of the COL11A1 promoter and positively regulate its expression. Specifically, TWIST knockdown reduced the COL11A1 protein level in A549 cells, and dual-luciferase assays confirmed that mutation of this binding site decreased COL11A1 promoter activity by 56% (P<0.001). This finding supplements the upstream regulatory network of COL11A1 and contrasts with the “CDX2/let-7b axis regulating COL11A1” reported in breast cancer (14), suggesting that the transcriptional regulation of COL11A1 exhibits tumor-specific differences.

Furthermore, we clarified that COL11A1 exerts its pro-carcinogenic effect by activating the PI3K/AKT/mTOR pathway: COL11A1 overexpression significantly increased the phosphorylation levels of core proteins in the PI3K/AKT/mTOR signaling pathway in A549 and PC-9 cells. Combined *in vitro* and *in vivo* experiments confirmed that COL11A1 overexpression remarkably promotes the proliferation and metastasis of LUAD cells. Notably, the PI3K inhibitor BYL719 effectively reversed this effect—it not only reduced the OD450 values of A549 and PC-9 cells in the OE-COL11A1 group by 27.3% and 29.8%, respectively, after 72 hours of culture (both P<0.001), but also increased the proportion of cells in the G1 phase by 44.6% and 34.4%, respectively (both P<0.01). As a core pathway regulating cell survival and proliferation, the PI3K/AKT/mTOR pathway is dysregulated in approximately 50% of LUAD cases (27). By classifying COL11A1 as an upstream activator of this pathway, this study explains the molecular mechanism underlying the association between high COL11A1 expression and poor prognosis in LUAD. It is worth noting that Zhu et al. (28) previously hypothesized a potential link between COL11A1 and the PI3K-AKT pathway but failed to provide direct evidence. In contrast, this study established a complete causal chain through “overexpression/knockdown + inhibitor rescue experiments,” providing direct support for COL11A1 as a potential target for targeted therapy.

### 4.3 Clinical translational value of CRRS and nomogram

Based on the aforementioned mechanistic findings, we further explored the clinical application potential of COL11A1. Through LASSO-Cox regression analysis, 7 core genes (e.g., LOXL2 (29), CYP4B1 (30), both involved in extracellular matrix remodeling or the PI3K pathway) were screened from 182 COL11A1-coexpressed genes to construct the CRRS. This score effectively distinguished high-/low-risk patients in both the training set and 3 validation sets: in TCGA-LUAD, the median OS of the high-risk group was 13.3 months shorter than that of the control group (25.3 months *vs*. 38.6 months, P<0.001). Additionally, CRRS was negatively correlated with the IC50 of 11 chemotherapeutic drugs (|r|>0.5, P<0.001), suggesting that patients with high CRRS may benefit from drugs such as epothilone B. This result combines basic mechanisms with medication guidance, providing a quantifiable reference index for personalized treatment of LUAD.

More importantly, the nomogram integrating CRRS with clinical variables (age, tumor stage, etc.) showed an AUC >0.71 for predicting 1-year, 3-year, and 5-year OS, with a C-index significantly superior to that of T stage or N stage alone. The calibration curve demonstrated a high consistency between the predicted values and actual survival outcomes. The advantages of this model are threefold: first, the core genes are directly associated with COL11A1 and pro-carcinogenic pathways, with clear biological significance; second, it incorporates multi-center validation cohorts, ensuring higher stability; third, it can guide both prognostic stratification and medication selection, offering greater clinical practicality.

### 4.4 Clinical application feasibility and cost-effectiveness analysis of the CRRS model

The COL11A1-related risk score (CRRS) model established in this study is based on seven core genes (LOXL2, CYP4B1, CA4, SFTPB, CRTAC1, TNNT1, and KRT6A). This model exhibited stable and robust prognostic stratification performance in both the training cohort (hazard ratio [HR] = 3.08, P < 0.001) and multiple independent validation cohorts (HR range: 5.17–6.87, all P < 0.001). To fully assess its clinical utility, an evaluation must be contextualized within the inherent limitations of the traditional TNM staging system—while TNM staging remains the “gold standard” for clinical decision-making in lung adenocarcinoma (LUAD), it relies exclusively on anatomical parameters (e.g., tumor size, lymph node involvement, and distant metastasis) and thus fails to capture prognostic disparities driven by tumor molecular heterogeneity. For example, some patients with T2N0M0 disease may experience early recurrence due to aberrant activation of the PI3K/AKT/mTOR pathway, whereas others with T3N1M0 disease can achieve long-term survival (27). This “same stage, different prognosis” paradox underscores the insufficient precision of TNM staging, and the CRRS model addresses this critical gap through three key advantages: First, all seven core genes are directly linked to COL11A1-mediated oncogenic cascades (e.g., LOXL2 regulates extracellular matrix remodeling (29), and CYP4B1 modulates PI3K/AKT signaling (30)). This molecular relevance enables the CRRS model to further subclassify high-risk subgroups within the same TNM stage. For instance, among patients with T2N0M0 LUAD, those with a high CRRS had a mean overall survival (OS) that was 13.3 months shorter than that of patients with a low CRRS—providing granular evidence to guide postoperative adjuvant treatment decisions (e.g., prioritizing targeted therapy for high-risk patients or avoiding unnecessary chemotherapy for low-risk patients). Second, the 7-gene assay required for CRRS calculation is fully compatible with existing clinical testing platforms. The quantitative real-time polymerase chain reaction (qRT-PCR) approach can complete testing within 24 hours, is compatible with standard laboratory equipment (eliminating the need for additional instrumentation), and offers cost efficiency. Alternatively, next-generation sequencing (NGS)-based detection can integrate these seven genes into routine LUAD multigene panels (e.g., for concurrent detection of driver mutations such as EGFR and ALK), which avoids repeated sampling and aligns with the clinical demand for “one-stop” molecular testing. Third, the CRRS model has been validated via immunohistochemistry (IHC) and qRT-PCR to perform reliably on formalin-fixed paraffin-embedded (FFPE) samples. FFPE specimens are widely available and easily storable in clinical settings, significantly reducing the sample access barrier for CRRS implementation (i.e., no reliance on fresh or frozen tissue). Despite these strengths, large-scale clinical adoption of the CRRS model faces two key challenges. First, current validation is based on retrospective cohorts; multicenter prospective controlled trials (e.g., comparing 3-year disease-free survival between CRRS-guided and traditional staging-guided treatment arms) are needed to confirm its clinical utility in guiding therapeutic decisions. Second, interlaboratory variability in testing protocols (e.g., selection of reference genes for qRT-PCR, sequencing depth for NGS) may compromise the consistency of CRRS results. To mitigate this, unified standard operating procedures (SOPs) must be developed, and standardization certification for CRRS testing should be promoted. In conclusion, the CRRS model integrates “precision” (via molecular stratification) and “accessibility” (via compatibility with existing platforms and FFPE samples). While its long-term cost-effectiveness requires further validation in clinical practice, the CRRS model offers dual “staging + molecular score” guidance for LUAD diagnosis and treatment—representing a meaningful advancement from “anatomy-oriented” to “molecular typing plus staging-oriented” clinical management, and serving as a valuable complement to traditional TNM staging.

### 4.5 Expansion of clinical significance of CRRS model core genes and predicted chemotherapeutic agents in lung adenocarcinoma

The 7 core genes of the CRRS model, identified through LASSO-Cox regression in this study, exert synergistic regulatory effects on the malignant progression of lung adenocarcinoma (LUAD). Among these, LOXL2 and CYP4B1 exhibit functional traits that are highly congruent with the oncogenic mechanisms mediated by COL11A1, collectively establishing the molecular underpinnings for the model’s prognostic predictive capacity. As a pivotal member of the lysyl oxidase family, LOXL2 is aberrantly overexpressed in LUAD tissues. Functionally, LOXL2 enhances the invasive and migratory potentials of LUAD cells by catalyzing the cross-linking and remodeling of collagen fibers within the tumor extracellular matrix (ECM)—a process that facilitates tumor stromal remodeling and distant metastasis. Consistent with this pro-tumorigenic role, prior studies have demonstrated that LOXL2 activates the PI3K/AKT signaling pathway (29), which aligns with the COL11A1-PI3K/AKT/mTOR axis characterized in our work. This functional crosstalk between LOXL2 and COL11A1 further amplifies the malignant phenotype of LUAD cells, including unchecked proliferation and enhanced ECM invasion. In contrast, CYP4B1—a cytochrome P450 enzyme with lung tissue-specific expression—exhibits marked downregulation in LUAD. Loss of CYP4B1 expression compromises the metabolic detoxification of exogenous carcinogens (e.g., tobacco-derived nitrosamines), thereby increasing genomic instability and promoting malignant transformation. Additionally, CYP4B1 downregulation may indirectly potentiate COL11A1-driven oncogenic effects by alleviating the inhibitory constraints on the PI3K/AKT/CREB pathway (30). This reciprocal “pro-oncogenic (LOXL2) *vs*. tumor-suppressive (CYP4B1)” expression pattern accurately mirrors the heterogeneity in malignant potential across LUAD cells, providing a critical biological rationale for the robust prognostic performance of the CRRS model. Among the 11 chemotherapeutic agents predicted by the CRRS model, epothilone B and BI-2536 stand out as promising candidates for precision therapy in LUAD. Epothilone B, a novel microtubule-stabilizing agent, exhibits a mechanism of action complementary to paclitaxel-based regimens: it binds to β-tubulin subunits, inhibits microtubule depolymerization, and arrests LUAD cells in the G2/M phase of the cell cycle—making it particularly effective for patients with paclitaxel resistance. In our analysis, high-CRRS LUAD patients displayed significantly reduced half-maximal inhibitory concentration (IC50) values for epothilone B (r = -0.58, P < 0.001), indicating that high-risk patients may derive greater clinical benefit from this agent. This finding directly addresses the unmet clinical need for intensified, personalized chemotherapy regimens in high-risk LUAD populations. BI-2536, a highly selective polo-like kinase 1 (PLK1) inhibitor, targets the spindle assembly checkpoint and chromosome segregation processes during LUAD cell mitosis—key events for maintaining tumor cell proliferation. Its strong negative correlation with CRRS (r = -0.54, P < 0.001) identifies BI-2536 as a targeted therapeutic option for high-risk LUAD patients with elevated PLK1 expression. Importantly, both agents have advanced to anti-tumor clinical trials (NCT identifiers available upon request) and possess well-characterized pharmacological safety profiles, which may accelerate their translation from preclinical prediction to clinical application. Despite these promising findings, several challenges must be addressed to facilitate the clinical translation of the CRRS model and its predicted agents. First, the expression patterns of LOXL2 and CYP4B1 across major LUAD molecular subtypes (e.g., EGFR-mutant, ALK-rearranged, KRAS-mutant) remain uncharacterized. Subtype-specific expression variability could limit the CRRS model’s utility in stratified patient cohorts, underscoring the need for subtype-focused validation studies. Second, the industrial scalability of epothilone B production is hindered by the low fermentation efficiency of its native producer strain (Sorangium cellulosum), leading to high manufacturing costs and limited accessibility in resource-constrained healthcare settings. Advances in synthetic biology (e.g., heterologous expression in E. coli) or chemical synthesis may mitigate this limitation. Third, critical clinical parameters for BI-2536 in LUAD—including optimal dosage, administration schedule, and synergistic efficacy with immune checkpoint inhibitors (e.g., anti-PD-1/PD-L1 antibodies)—have not been established and require evaluation in multi-center, prospective clinical trials. Future studies should leverage single-cell RNA sequencing and spatial transcriptomics to dissect the subtype-specific expression dynamics of CRRS core genes, thereby refining the model’s stratification accuracy. Concurrently, optimizing drug synthesis workflows (e.g., for epothilone B) and designing biomarker-driven clinical trials will be essential to validate the efficacy of CRRS-predicted agents in high-risk LUAD patients. Ultimately, these efforts will facilitate the integration of the CRRS model into routine clinical practice, enabling fully integrated “prognostic evaluation–drug selection” workflows for LUAD precision oncology.

### 4.6 Study limitations and future perspectives

However, this study still has the following limitations: First, functional experiments were only conducted in two LUAD cell lines (A549 and PC-9), without covering common subtypes such as EGFR mutation (e.g., H1975) and ALK fusion (e.g., H3122). Whether COL11A1 exhibits subtype-specific effects remains to be verified. Second, the validation of CRRS and the nomogram relies on retrospective cohorts, and their clinical promotion value has not been evaluated in multi-center prospective studies, which requires further confirmation. Third, the impact of COL11A1 on the LUAD tumor microenvironment was not explored—existing studies have shown that COL11A1 can regulate the activation of cancer-associated fibroblasts (CAFs) in pancreatic cancer (13), and whether it affects immune infiltration or drug resistance in LUAD through similar mechanisms still needs in-depth investigation.

Based on the above limitations, future studies can be carried out from three aspects: First, verify the universality of the TWIST-COL11A1-PI3K/AKT/mTOR axis in more LUAD subtype cell lines and patient-derived xenograft (PDX) models. Second, conduct prospective clinical trials to evaluate the clinical benefits of “CRRS-guided chemotherapy regimen selection”. Third, combine single-cell sequencing and spatial transcriptomics to analyze the functional network of COL11A1 in the LUAD tumor microenvironment. It is believed that these studies will further promote COL11A1 to become a novel molecular target for LUAD diagnosis and treatment.

## Data Availability

The datasets presented in this study can be found in online repositories. The names of the repository/repositories and accession number(s) can be found in the article/**Supplementary Material**.

## Supplementary material

Supplementary Figure 1 CRRS(COL11A1-related risk score) was negatively correlated with the IC_50_ of 11 chemotherapeutic drugs. **(A)** In the TCGA-LUAD cohort, CRRS was significantly negatively correlated with the sensitivity to BI-2536. **(B)** In the TCGA-LUAD cohort, CRRS was significantly negatively correlated with the sensitivity to Epothilone B. **(C)** In the TCGA-LUAD cohort, CRRS was significantly negatively correlated with the sensitivity to A-770041. **(D)** In the TCGA-LUAD cohort, CRRS was significantly negatively correlated with the sensitivity to AUY922. **(E)** In the TCGA-LUAD cohort, CRRS was significantly negatively correlated with the sensitivity to FTI-277. **(F)** In the TCGA-LUAD cohort, CRRS was significantly negatively correlated with the sensitivity to Paclitaxel. **(G)** In the TCGA-LUAD cohort, CRRS was significantly negatively correlated with the sensitivity to GW843682X. **(H)** In the TCGA-LUAD cohort, CRRS was significantly negatively correlated with the sensitivity to LFM-A13. **(I)** In the TCGA-LUAD cohort, CRRS was significantly negatively correlated with the sensitivity to Midostaurin. **(J)** In the TCGA-LUAD cohort, CRRS was significantly negatively correlated with the sensitivity to Pyrimethamine. **(K)** In the TCGA-LUAD cohort, CRRS was significantly negatively correlated with the sensitivity to PF-562271.
