# Supplementary figures and images for: COL11A1 promotes lung adenocarcinoma progression via PI3K/AKT/mTOR pathway: mechanistic insights and development of a COL11A1-related prognostic signature
Source: Front Oncol. 2026 Feb 27;16:1748723. doi: 10.3389/fonc.2026.1748723 (PMC12982051; doi:10.3389/fonc.2026.1748723)

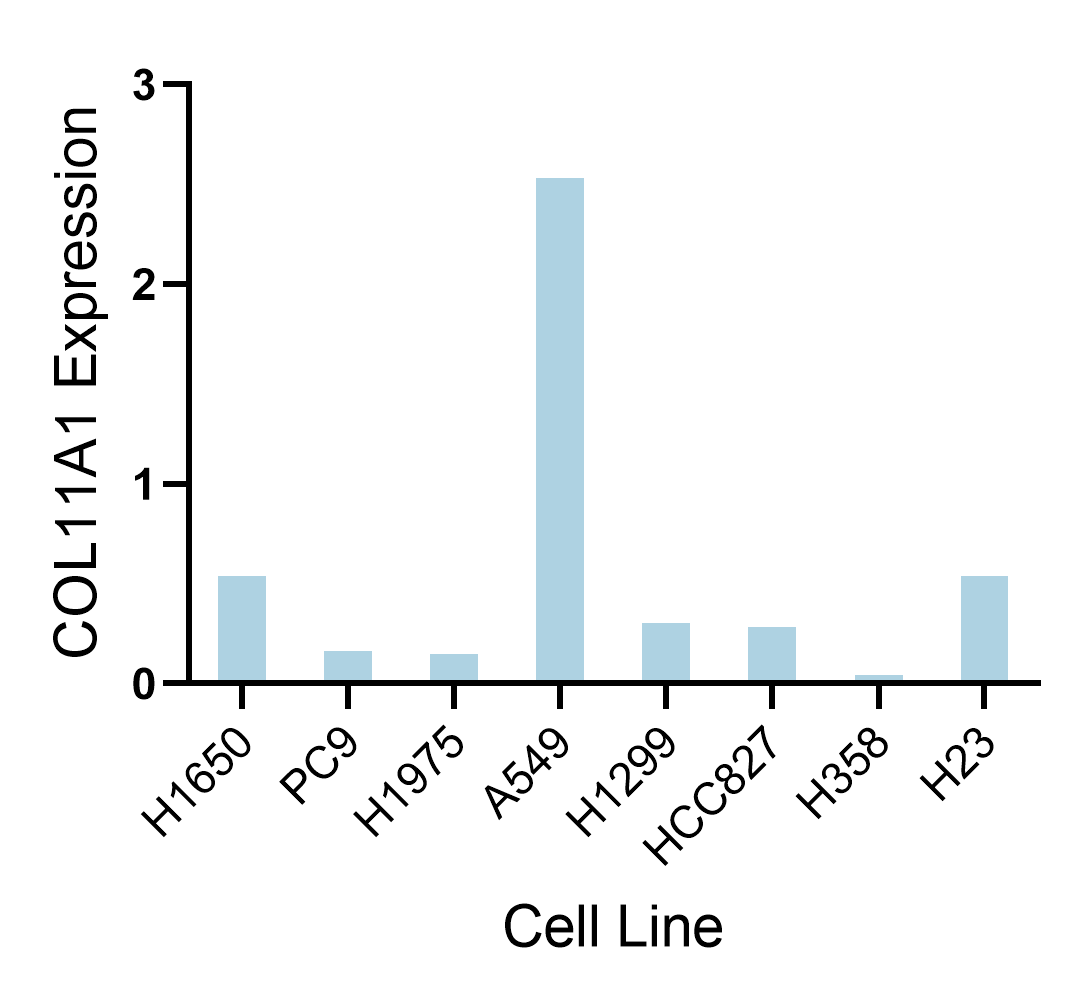

Supplement: Supplementary Figure 1 — CRRS(COL11A1-related risk score) was negatively correlated with the IC50 of 11 chemotherapeutic drugs. (A) In the TCGA-LUAD cohort, CRRS was significantly negatively correlated with the sensitivity to BI-2536. (B) In the TCGA-LUAD cohort, CRRS was significantly negatively correlated with the sensitivity to Epothilone B. (C) In the TCGA-LUAD cohort, CRRS was significantly negatively correlated with the sensitivity to A-770041. (D) In the TCGA-LUAD cohort, CRRS was significantly negatively correlated with the sensitivity to AUY922. (E) In the TCGA-LUAD cohort, CRRS was significantly negatively correlated with the sensitivity to FTI-277. (F) In the TCGA-LUAD cohort, CRRS was significantly negatively correlated with the sensitivity to Paclitaxel. (G) In the TCGA-LUAD cohort, CRRS was significantly negatively correlated with the sensitivity to GW843682X. (H) In the TCGA-LUAD cohort, CRRS was significantly negatively correlated with the sensitivity to LFM-A13. (I) In the TCGA-LUAD cohort, CRRS was significantly negatively correlated with the sensitivity to Midostaurin. (J) In the TCGA-LUAD cohort, CRRS was significantly negatively correlated with the sensitivity to Pyrimethamine. (K) In the TCGA-LUAD cohort, CRRS was significantly negatively correlated with the sensitivity to PF-562271. [file Image1.png]
