# Supplementary material for: COL11A1 promotes lung adenocarcinoma progression via PI3K/AKT/mTOR pathway: mechanistic insights and development of a COL11A1-related prognostic signature
Source: Front Oncol. 2026 Feb 27;16:1748723. doi: 10.3389/fonc.2026.1748723 (PMC12982051; doi:10.3389/fonc.2026.1748723)
Supplement: Supplementary file 2 [file DataSheet1.docx]

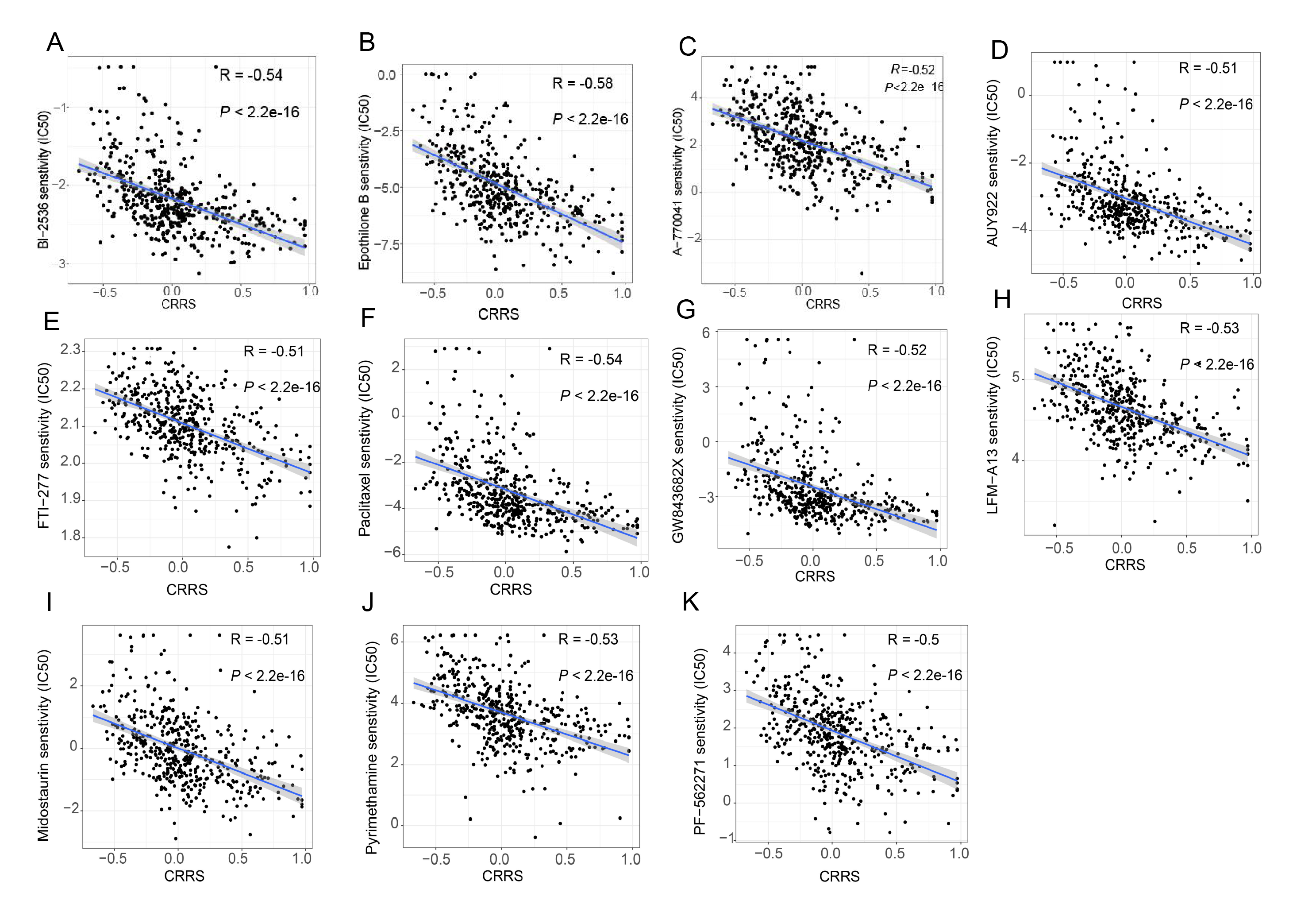


**Supplementary figure 1. CRRS(COL11A1-related risk score) was negatively correlated with the IC₅₀ of 11 chemotherapeutic drugs.**

(A) In the TCGA-LUAD cohort, CRRS was significantly negatively correlated with the sensitivity to BI-2536.(B) In the TCGA-LUAD cohort, CRRS was significantly negatively correlated with the sensitivity to Epothilone B.(C) In the TCGA-LUAD cohort, CRRS was significantly negatively correlated with the sensitivity to A-770041.(D) In the TCGA-LUAD cohort, CRRS was significantly negatively correlated with the sensitivity to AUY922.(E) In the TCGA-LUAD cohort, CRRS was significantly negatively correlated with the sensitivity to FTI-277.(F) In the TCGA-LUAD cohort, CRRS was significantly negatively correlated with the sensitivity to Paclitaxel.(G) In the TCGA-LUAD cohort, CRRS was significantly negatively correlated with the sensitivity to GW843682X.(H) In the TCGA-LUAD cohort, CRRS was significantly negatively correlated with the sensitivity to LFM-A13.(I) In the TCGA-LUAD cohort, CRRS was significantly negatively correlated with the sensitivity to Midostaurin.(J) In the TCGA-LUAD cohort, CRRS was significantly negatively correlated with the sensitivity to Pyrimethamine.(K) In the TCGA-LUAD cohort, CRRS was significantly negatively correlated with the sensitivity to PF-562271.
