# Supplementary material for: COL11A1 promotes lung adenocarcinoma progression via PI3K/AKT/mTOR pathway: mechanistic insights and development of a COL11A1-related prognostic signature
Source: Front Oncol. 2026 Feb 27;16:1748723. doi: 10.3389/fonc.2026.1748723 (PMC12982051; doi:10.3389/fonc.2026.1748723)
Supplement: Supplementary file 3 [file Table1.docx]

| COL11A1 |
| --- |
| CLMP |
| SHOX2 |
| PRRX1 |
| PDGFRA |
| KIF26B |
| IGFBP3 |
| FMO1 |
| RUNX2 |
| CCN4 |
| ITGA11 |
| SYNDIG1 |
| VEGFD |
| COL1A2 |
| TMEM158 |
| ADAMTS16 |
| WNT2 |
| COL6A1 |
| P4HA3 |
| CLEC11A |
| GAS1 |
| HEPH |
| SFRP4 |
| NID2 |
| LAMA4 |
| SCT |
| SFRP2 |
| TENM3 |
| ITGB5 |
| SEMA5B |
| ASPN |
| ADAM12 |
| PLPP4 |
| COL5A1 |
| FBXO32 |
| MFAP2 |
| TWIST1 |
| HTRA1 |
| SULF2 |
| POSTN |
| SGCD |
| LOX |
| FRMD6 |
| SLC2A5 |
| SPOCD1 |
| ADAMTS5 |
| BGN |
| LOXL1 |
| APCDD1L |
| HS3ST3A1 |
| ANTXR1 |
| BMP8A |
| FIBIN |
| SLC12A8 |
| TNFSF4 |
| TGFBI |
| VCAM1 |
| GPC6 |
| CCL26 |
| ADAMTS12 |
| TAGLN |
| FBLN2 |
| COL10A1 |
| ARSI |
| INHBA |
| ADAMTS2 |
| CALD1 |
| ITGA5 |
| MMP3 |
| UNC5B |
| COL1A1 |
| LUM |
| SPARC |
| ANGPTL2 |
| CALU |
| DACT1 |
| COL12A1 |
| PCOLCE |
| PLAU |
| COL5A2 |
| PDLIM7 |
| RCN3 |
| SULF1 |
| OMD |
| SPHK1 |
| UBE2QL1 |
| GREM1 |
| POGLUT2 |
| FNDC1 |
| CERCAM |
| GFPT2 |
| CMTM3 |
| MFAP5 |
| ZNF469 |
| CPXM1 |
| RFLNA |
| THBS2 |
| GPR176 |
| GLT8D2 |
| CCL11 |
| MMP2 |
| PGF |
| BNC2 |
| PALLD |
| NUDT11 |
| ISLR |
| CILP |
| SUGCT |
| RASGRF2 |
| P3H1 |
| FAP |
| LOXL2 |
| IL11 |
| MEDAG |
| PDPN |
| BICC1 |
| SERPINH1 |
| ROR2 |
| THY1 |
| EMILIN1 |
| LRRC15 |
| GJB2 |
| MRGPRF |
| COL5A3 |
| IBSP |
| MMP11 |
| PLN |
| CRISPLD2 |
| COL8A1 |
| ALDH1B1 |
| MN1 |
| FBN1 |
| CTHRC1 |
| CILP2 |
| ENPP1 |
| FSTL1 |
| TGFB3 |
| SNAI2 |
| SPOCK1 |
| LGALS1 |
| C1QTNF6 |
| TIMP2 |
| MMP14 |
| VCAN |
| TNFAIP6 |
| CDH13 |
| RARRES2 |
| FN1 |
| COL6A2 |
| CALHM5 |
| GXYLT2 |
| RGS4 |
| GPX8 |
| PRR16 |
| EPYC |
| AEBP1 |
| NOX4 |
| ALDH1L2 |
| COMP |
| ENOX1 |
| CCDC80 |
| MEIS3 |
| NTM |
| BEND6 |
| NPTX2 |
| CTSK |
| CCDC71L |
| COL8A2 |
| DIO2 |
| TSHZ3 |
| ACTA2 |
| COL15A1 |
| COL3A1 |
| HTRA3 |
| OLFML2B |
| ACTN1 |
| PTPRN |
| MXRA5 |
| MSC |
| ADAMTS4 |
| COL6A3 |
| KCND2 |
