# Supplementary material for: COL11A1 promotes lung adenocarcinoma progression via PI3K/AKT/mTOR pathway: mechanistic insights and development of a COL11A1-related prognostic signature
Source: Front Oncol. 2026 Feb 27;16:1748723. doi: 10.3389/fonc.2026.1748723 (PMC12982051; doi:10.3389/fonc.2026.1748723)
Supplement: Supplementary file 4 [file Table2.docx]

| COL11A1 |
| --- |
| PLPP4 |
| VEGFD |
| TWIST1 |
| ADAM12 |
| SPOCK1 |
| CTHRC1 |
| TMEM158 |
| APCDD1L |
| COL12A1 |
| RFLNA |
| LOXL2 |
| SULF1 |
| GREM1 |
| GJB2 |
| THBS2 |
| CYP4B1 |
| LRRC15 |
| COL3A1 |
| ADH1B |
| COL1A1 |
| ITGA11 |
| CACNA2D2 |
| MFAP5 |
| INMT |
| CA4 |
| PEBP4 |
| SFRP2 |
| PLA2G1B |
| MMP11 |
| CCL11 |
| FNDC1 |
| AGER |
| LGI3 |
| EPYC |
| SFTPC |
| C16orf89 |
| GKN2 |
| AQP4 |
| WIF1 |
| SCGB3A2 |
| CLDN18 |
| PGC |
| SUSD2 |
| SFTPD |
| LRRK2 |
| SOSTDC1 |
| SFTPB |
| CILP |
| MMP12 |
| CPB2 |
| FOLR1 |
| ATP13A4 |
| ZNF385B |
| C7 |
| SLC26A9 |
| CHIA |
| SPP1 |
| CRTAC1 |
| GGTLC1 |
| SCGB3A1 |
| VSIG2 |
| NAPSA |
| SCTR |
| SLC22A31 |
| MMP1 |
| SFTPA2 |
| SFTPA1 |
| MS4A15 |
| AGTR2 |
| KRT16 |
| C4BPA |
| GJB1 |
| TNNT1 |
| MYEOV |
| PIGR |
| DMBT1 |
| PCSK2 |
| SCGB1A1 |
| AQP5 |
| KRT6A |
